# Genomics and genetics of *Sulfolobus islandicus* LAL14/1, a model hyperthermophilic archaeon

**DOI:** 10.1098/rsob.130010

**Published:** 2013-04

**Authors:** Carole Jaubert, Chloë Danioux, Jacques Oberto, Diego Cortez, Ariane Bize, Mart Krupovic, Qunxin She, Patrick Forterre, David Prangishvili, Guennadi Sezonov

**Affiliations:** 1 Département de Microbiologie, Unité Biologie Moléculaire du Gène chez les Extrêmophiles, Institut Pasteur, Paris, France; 2 CNRS, UMR8621, Institut de Génétique et Microbiologie, Université Paris-Sud 11, 91405 Orsay Cedex, France; 3 Irstea, UR HBAN, 92761 Antony, France; 4 Danish Archaea Centre, Department of Biology, University of Copenhagen, Ole Maaløes Vej 5, 2200 Copenhagen N, Denmark; 5 UMR 7138 ‘Systématique, Adaptation, Evolution’, Université Pierre et Marie Curie, Paris, France

**Keywords:** Archaea, *Sulfolobus islandicus* LAL14/1, genome analysis, genetics, CRISPR

## Abstract

The 2 465 177 bp genome of *Sulfolobus islandicus* LAL14/1, host of the model rudivirus SIRV2, was sequenced. Exhaustive comparative genomic analysis of *S. islandicus* LAL14/1 and the nine other completely sequenced *S. islandicus* strains isolated from Iceland, Russia and USA revealed a highly syntenic common core genome of approximately 2 Mb and a long hyperplastic region containing most of the strain-specific genes. In LAL14/1, the latter region is enriched in insertion sequences, CRISPR (clustered regularly interspaced short palindromic repeats), glycosyl transferase genes, toxin–antitoxin genes and MITE (miniature inverted-repeat transposable elements). The tRNA genes of LAL14/1 are preferential targets for the integration of mobile elements but clusters of atypical genes (CAG) are also integrated elsewhere in the genome. LAL14/1 carries five CRISPR loci with 10 per cent of spacers matching perfectly or imperfectly the genomes of archaeal viruses and plasmids found in the Icelandic hot springs. Strikingly, the CRISPR_2 region of LAL14/1 carries an unusually long 1.9 kb spacer interspersed between two repeat regions and displays a high similarity to pING1-like conjugative plasmids. Finally, we have developed a genetic system for *S. islandicus* LAL14/1 and created *ΔpyrEF* and *ΔCRISPR_1* mutants using double cross-over and pop-in/pop-out approaches, respectively. Thus, LAL14/1 is a promising model to study virus–host interactions and the CRISPR/Cas defence mechanism in Archaea.

## 2. Introduction

The genus *Sulfolobus* was first described by Brock *et al*. in 1972 [1] and includes thermoacidophilic Archaea that grow at 70–85°C and pH 2–3 under aerobic conditions either chemolithotrophically by oxidizing elementary sulfur/hydrogen sulfide or heterotrophically [2]. *Sulfolobus* strains have been isolated from various acidic thermal habitats (in the USA, Italy, Iceland, Russia and elsewhere). They are easily maintained under laboratory conditions, making them convenient models to study the molecular organization of the archaeal cell [3].

The sequences of 12 *Sulfolobus* genomes are currently available. They include *Sulfolobus solfataricus* P2 [4], *Sulfolobus tokodaii* [5], *Sulfolobus acidocaldarius* [6] and nine strains of *Sulfolobus islandicus*: HV10/4 and REY15A [7] (isolated from hot springs in Iceland) [8]; M.14.25, M.16.27 and M.16.4 (from hot springs at the Mutnovsky Volcano, Kamchatka, Russia); Y.N.15.51 and Y.G.57.14 (from hot springs in Yellowstone National Park, USA); and L.D.8.5 and L.S.2.15 (from Lassen National Park, USA) [9].

The strain *S. islandicus* LAL14/1 was isolated in 1995 from a solfataric field in Iceland by the group of Zillig [8]. Its geographical origin, growth requirements and physiology indicate that LAL14/1 is a close relative of two *S. islandicus* strains also isolated from Iceland, HVE10/4 and REY15A. However, LAL14/1 has a particular pattern of sensitivity to various archaeal viruses. LAL14/1 is resistant to the rudivirus SIRV1 but can be efficiently infected by its close relative SIRV2 [10], which has a complex cycle of development in the host cells. At the end of the infection cycle, that lasts about 14 h, specific pyramid-like structures are formed on the cell surface facilitating release of virus particles [11–13]. These unique characteristics make *S. islandicus* LAL14/1 an interesting model to study virus–host interactions in Archaea.

Effective genetic tools have been developed for a limited number of *Sulfolobus* species [14], including *S. solfataricus* P1 and 98/2 [15,16], *S. acidocaldarius* [17,18], *S. islandicus* REY15A [19–21] and *S. islandicus* M.16.4 [22]. However, genetic approaches have not previously been available for LAL14/1.

In this study, we report the results of the *in silico* analysis of the genome sequence of *S. islandicus* LAL14/1 and detailed comparisons with other available *S. islandicus* strains, and in particular the closely related strains, REY15A and HVE10/4. We also have established genetic tools for this strain by creating both *ΔpyrEF* and *ΔCRISPR_1* mutants. This work has made substantial progress towards the possibility of applying powerful global approaches (for example, transcriptome, RNAseq and proteome analyses) to elucidate the interplay between host and viral genes and proteins during the viral infection cycle.

## 3. Material and methods

### 3.1. Strains growth

*Sulfolobus islandicus* strains were grown aerobically at 80°C and under constant agitation in rich medium containing 0.2 g l^−1^ of Tryptone Peptone, 2 g l^−1^ of sucrose and 1 g l^−1^ of yeast extract. The minimal medium used to select Ura^+^ variant isolates was as described previously [2]. Ura^−^ mutants were selected on rich solid medium in the presence of 50 mg l^−1^ of 5′-fluoroorotic acid.

### 3.2. Genetic experiment

#### 3.2.1. PCR amplification

*pyrEF mutant.* The following primers were used to amplify the locus, including the *pyrEF* operon and the upstream (1 kb) and downstream (1 kb) situated regions of the *S. islandicus* E233S chromosome: oligoUP, CAGTAGCTAAAACAATTGAAAGAGTAGGTG; oligoDOWN, CTAATGATGCTTGATAGAAGTATTTAGCGT. The PCR amplification was performed in 50 µl of reaction mixture containing 10 µM of each primer, 1 µl template, 10 µl 5× HF Phusion Buffer (Finzyme), 10 nM dNTPs and 0.5 µl *pfu* DNA polymerase (Finzyme) with the following conditions: 30 s at 98°C, 60 s at 55°C and 90 s at 72°C for 35 cycles.

*CRISPR mutant.* The following primers were used to amplify the DNA fragments IN (oligoup1, AAAAAACCATGGTACGATTCCGCTTAAGCC; oligodown2, AAAAAAGGATCCGTAATGAGAGCTTGGTTT); OUT (oligoup3, AAAAAGTCGACTACTACCGTGTACTTCCCC; oligodown4, AAAAACCATGGTGCGTTAATGAGGCAAGGT) and TARGET (oligoup5, AAAAAAGCATGCTTCTGCTCAAAAGGAGGA; oligodown6, AAAAAACTGCAGTAGAAGAAGATAGCCCAC). The positions of these fragments is indicated in figure 8.

*Transformation*. Electroporation of *S. islandicus* LAL14/1 was performed as described by Deng *et al*. [19].

### 3.3. Genome sequencing

Total DNA was extracted from the cells using phenol–chloroform and ethanol precipitation. The sequencing was done by Fidelity System Inc. using Illumina technology and assembled using the software Velvet v. 1.2 [23]. The genome was automatically annotated and refined manually. Open reading frames (ORFs) were predicted using *phred*, *phrap* and *consed* software [24–26] and tRNA with tRNAscan-SE [27]. Putative insertion sequence (IS) elements were identified by BLASTn search against the IS Finder Database (http://www.is-biotoul.fr/). Annotations were manually curated using Ugene software [28].

### 3.4. Phylogenetic analysis

The genome sequences of nine *S. islandicus* strains were downloaded as Genbank files from the NCBI database (NC_012588, NC_012623, NC_017275, NC_013769, NC_012589, NC_012632, NC_012726, NC_017276 and NC_012622).

The DNA sequences of all genomes were aligned using the *progressiveMauve* algorithm with default parameters and analysed with *stripSubsetLCBs*. The *S. islandicus* core genome sequence was used for phylogenetic dating based on the standard rate of accumulation of random mutations in hyperthermophilic Archaea (4.66 × 10^−9^ substitution per site per year; [9]). Clonal genealogy was inferred using the *ClonalFrame* algorithm three times.

### 3.5. Dot-Plot

The Ugene Dot-Plot algorithm with the option ‘search for inverted repeats’ enabled was used to generate dot-plots from pairs of sequences. Sequences from the 10 *S. islandicus* strains were aligned with the *progressiveMauve* algorithm with scoring parameters divided by four to ensure the recognition of large conserved regions.

### 3.6. Replication origins

Zplotter applet v. 2.0 was used to calculate Zcurves (http://tubic.tju.edu.cn/zcurve/).

OriC1 and OriC2 structures were determined by alignment of OriC1 and OriC2 sequences in *S. solfataricus* P2 with *S. islandicus* LAL14/1 sequences using the BLASTn algorithm with default parameters. OriC3 structures were determined by mapping UCM (UnCharacterized Motif) sequences in the genome of LAL14/1 using the Ugene search tool with default parameters.

### 3.7. Exceptional motifs

R'MES software [29] was used to evaluate the significance of motif frequency in *S. islandicus* genomes. This statistical method compares the observed count of each motif to the count predicted by a reference probabilistic Markovian model. Exceptional motifs of lengths two to eight were analysed choosing models according to the authors’ guidelines (http://migale.jouy.inra.fr/?q=method) and using the highest possible order each time (usually *I*-2, where *I* is the length of the studied motif).

### 3.8. Spacer data

All available CRISPR spacers from *S. islandicus* LAL14/1 were determined using CRISPRfinder (http://crispr.u-psud.fr/Server/) [30].

### 3.9. PAM motifs

To find PAM motifs, protospacers corresponding to the selected spacers listed in the electronic supplementary material, table S15 were aligned and visualized with WebLogo (http://weblogo.berkeley.edu/logo.cgi). For each protospacer, the regions analysed were 10 nucleotide-long sequences immediately upstream and downstream from the region identical or similar to the spacer.

### 3.10. *Sulfolobus islandicus* pan-genome characterization

The pan-genome of the 10 available *S. islandicus* strains was obtained with an in-house program [31]. Briefly, the proteins encoded by the 10 genomes were compared using two-way BLASTp analysis and ranked in families of orthologous proteins according to the criteria defined by Lerat *et al.* [32].

## 4. Results

### 4.1. General features of the *Sulfolobus islandicus* LAL14/1 genome

The genome of *S. islandicus* strain LAL14/1 (NCBI accession no. CP003928) was sequenced at 104-fold coverage by Fidelity System (http://fidelitysystems.com/) using Illumina technology. The protein-coding genes were annotated using Ugene software [28]. The *S. islandicus* LAL14/1 genome consists of a single circular chromosome of 2 465 177 bp; 85.6 per cent of the genome is coding. The chromosome carries 2601 protein-coding genes and has a GC content of 35 per cent. The general properties of the *S. islandicus* LAL14/1 genome composition are summarized in table 1.

**Table 1.** General properties and composition of the genome of *S. islandicus* LAL14/1.

genome size	2 465 177 bp
protein-coding genes	2601 (85.6%)
CDS average size	815 bp
average size of intergenic regions	190 bp
tRNA-coding genes	45 functional tRNA

### 4.2. Phylogenetic position of *Sulfolobus islandicus* LAL14/1

**Figure 1.**
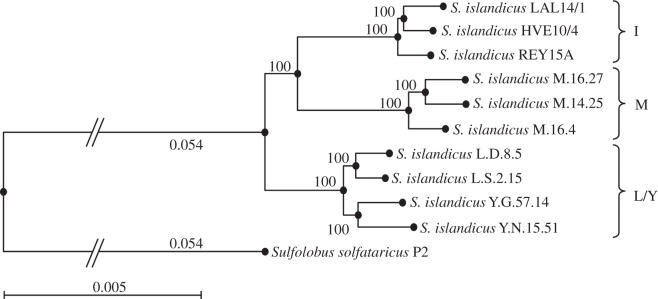
Phylogenetic position of *S. islandicus* LAL14/1 among other sequenced *S. islandicus* strains. *Sulfolobus solfataricus* P2 is used as an external group. The length of branches is proportional to the phylogenetic distance between the strains. Bootstrap values are indicated.

The phylogenetic tree of available *S. islandicus* strains was established by comparison of chromosomal DNA sequences shared by all 10 strains (figure 1) as described in §3. The strain LAL14/1 is phylogenetically very close to the strains HVE10/4 and REY15A, also isolated from Iceland. On the basis of the standard rate of random mutation accumulation in *S. islandicus* [9], we estimate that strain REY15A separated from the clade LAL + HVE about 460 000 years ago and strain LAL14/1 diverged from the clade LAL + HVE 60 000 years later. The tree is divided into three main clades corresponding to the geographical origins of the strains: clade I from Iceland; clade M from Kamchatka (Russia) and clade L/Y from Lassen and Yellowstone (USA) [9,33].

The general features of the 10 *S. islandicus* genomes are reported in table 2. LAL14/1 has the smallest genome, apparently due to the small number of horizontally transferred CAG regions (see below). It carries a remarkably complex CRISPR system, with more spacers than the other *S. islandicus* genomes, some matching the virus SIRV1 perfectly.

**Table 2.** Comparison of genomic patterns of 10 *S. islandicus* strains (compilation of our own data and those from [7,9]. CAG are discussed elsewhere in the text. SIRV1 and SIRV2 are rudiviruses described in [10,34,35]. R, resistant; S, sensitive; nd, no data. CRISPR and Cmr families are indicated following established classification [36,37].

	LAL14/1	REY15A	HVE10/4	L.D.8.5	L.S.2.15	M.16.4	M.16.27	M.14.25	Y.G.57.14	Y.N.15.51
genome size (Mb)	2.4	2.7	2.5	2.7	2.7	2.6	2.7	2.6	2.7	2.8
gene no.	2601	2666	2745	2939	2760	2759	2680	2632	2928	2868
CAG no.	3	4	7	16	13	5	5	11	16	22
CRISPR/Cas families (no. of spacers)	I (199) III (84)	I (208)	I (215) III (47)	I (223) III (42)	I (225) II (7)	I (139)	I (164) II (72)	I (143) II (39)	I (57)	I (99)
Cmr families	B,F	B	2 × B	B	B	2 × B	B	B,D	3 × B	E
perfect spacers against SIRV1 and phenotype	2 (R)	0 (R)	0 (R)	0 nd	0 nd	0 nd	0 nd	0 nd	0 nd	0 nd
perfect spacers against SIRV2 and phenotype	0 (S)	2 (R)	3 (R)	0 nd	0 nd	0 nd	0 nd	0 nd	0 nd	0 nd

### 4.3. The *Sulfolobus islandicus* pan-genome and specific genomic pattern of the strain LAL14/1

The first version of the *S. islandicus* pan-genome was published in 2009, based on the analysis of seven strains; it includes 20 610 proteins [9]. Three additional *S. islandicus* genome sequences have since become available (REY15A, HVE10/4 [7] and LAL14/1 (present work)). The new updated version of the *S. islandicus* pan-genome has 27 578 proteins (*in silico* prediction) that can be divided into 3492 families of orthologous proteins (see §3). The statistics of the family distribution is presented in the electronic supplementary material, figure S1. There are 1892 ubiquitous families, present in at least one copy in all of the *S. islandicus* genomes. This group, indicated in the annotation by *arCOG* + *number*, constitutes the *S. islandicus* core-genome.

There are 1030 families present in two or more (but not all) strains. The best-represented families of *S. islandicus* include various transposases, ABC transporters and CoA pathway genes (see the electronic supplementary material, table S1). The majority of these families are present in the LAL14/1 genome, and some are overrepresented (more frequent than predicted from the average pan-genome statistics), for example, the transposases belonging to the *IS*1 family. Others, for example transposases of families *IS*H3 and *IS*110, are clearly underrepresented.

There are 570 families classified as *singletons*. They are strictly strain-specific and present in only one copy per genome (see the electronic supplementary material, figure S2 and table S2). The *S. islandicus* LAL14/1 genome contains 65 singletons, and specific functions could be predicted for 14 of them. They include a putative transcription regulator of the MarR family (SiL_0405), a putative acyl-coenzyme A synthetase/AMP fatty acid ligase (SiL_0481), a small subunit of the methyltransferase (SiL_0587), a putative glycosyltransferase (SiL_0818), a membrane protein involved in the export of O-antigen and teichoic acid (SiL_0839), a putative secreted endonuclease distantly related to the archaeal Holliday junction resolvase (SiL_1319), an Fe–S oxidoreductase (SiL_1473), and seven families of Cmr proteins related to CRISPRs: Cmr3 (SiL_0600), Cas10 (SiL_0601), Cmr6 (SiL_0602), Cmr5 (SiL_0603), Cmr1 (SiL_0604), Cmr4 (SiL_0605) and Csm6-like protein (SiL_0630).

The taxonomical distribution of the individual genes and singletons of *S. islandicus* LAL14/1 is summarized in table 3. Of the 2601 annotated protein-coding genes, only 4.7 per cent are exclusive to this strain; 10 per cent are only found in *S. islandicus* species and 37.6 per cent are specific to Sulfolobales. The 51 *S. islandicus* LAL14/1-specific singletons, and the seven *S. islandicus*-specific and seven Crenarchaeota-specific singletons are listed in the electronic supplementary material, tables S3A–C.

**Table 3.** Taxonomic specificity of the protein-coding genes of *S. islandicus* LAL14/1.

gene specificity	*S. islandicus* LAL14/1	
	total gene distribution	singleton distribution
*S. islandicus* LAL14/1-exclusive	123	51
*S. islandicus*-exclusive (10 strains)	134	0
*Sulfolobus*-specific	719	7
Crenarchaeota-specific	238	7
Archaea-specific	409	0
Archaea + Bacteria-specific	535	0
Archaea + Eukarya specific	72	0
universal	371	0
total	2601	65

The specific genomic pattern of LAL14/1 is presented in table 4. Like all the other studied *S. islandicus* genomes, LAL14/1 codes for a large number of transposases representing families composed of multiple paralogues. In this strain, the most represented family is the protein OrfB encoded by IS*200*/IS*605*. The transposon ISC*1048* of the family IS*607* is also overrepresented. However, other transposase families, such as families IS*110* and IS*H3*, are clearly underrepresented in LAL14/1.

**Table 4.** Major groups of paralogues in *S. islandicus* LAL14/1 and their representation in 10 *S. islandicus* genomes.

*S. islandicus* LAL14/1		protein families	all *S. islandicus*	
range	protein no.		protein no.	range
1	16	IS200/*605* families protein OrfB^a^	129	3
2	13	oligopeptide/dipeptide ABC transporter, ATPase subunit^a^	113	4
3	10	IS*630* family transposase^a^	67	8
4	9	IS*1* family transposase	53	7
5	6	IS*110* family transposase^a^	151	1
6	6	3-hydroxyacyl-CoA dehydrogenase NAD-binding protein^a^	78	5
7	5	inosine/uridine nucleoside hydrolase	32	30
8	4	high-affinity nickel-transporter	21	68
9	4	dTDP-4-dehydrorhamnose 3,5-epimerase	18	145

^a^Includes several *nodes*.

**Figure 2.**
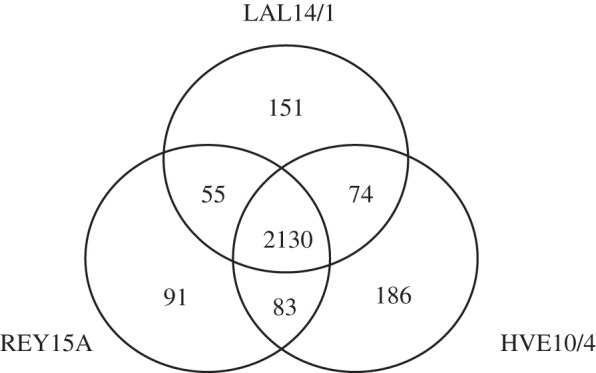
Conservation of protein *nodes* in three closely related *S. islandicus* strains. A total of 2130 families are shared by three strains. Each of the strain is characterized by the presence of a specific set of families: 151 for LAL14/1; 91 for REY15A and 186 for HVE10/4.

The families of orthologous proteins were compared between the *S. islandicus* strains LAL14/1, REY15A and HVE10/4, all isolated from the same geographical location. This revealed substantial similarity between the genomes of these strains: of the 2770 families analysed, 2130 (77%) are shared by these three *S. islandicus* genomes. A surprisingly large number of families are unique for each of these strains (figure 2), constituting a specific genomic signature for each of the strains.

### 4.4. Exceptional motifs in *Sulfolobus islandicus* LAL14/1 genome

Many non-coding motifs have specific biological functions in genomes and statistical analyses of oligomer frequencies in genome sequences can identify possibly significant motifs (e.g. reviewed in [38] for bacteria). Similar analyses have also been very useful for studying the evolution of genome functions and regulation [39].

Systematic analyses of short oligonucleotides of fixed composition (usually called *words*) were conducted with the LAL14/1 and other *S. islandicus* genomes to identify non-coding functional motif candidates. The *word* frequency and preference patterns are very similar in the three closely related *S. islandicus* strains, suggesting that their functional motifs and associated mechanisms are generally similar. Some more pronounced differences were observed for longer *words*, and this was mainly associated with the different genomic content of the large variable regions.

As commonly observed in archaeal and bacterial genomes, palindromic motifs are generally avoided (e.g. electronic supplementary material, table S4 for LAL14/1 and electronic supplementary material, table S5), possibly as a consequence of the presence of restriction–modification systems encoded in the genome [40] or of other biological phenomena such as the control of chromosome replication [38]. Among the analysed words (see the electronic supplementary material, tables S4 and S5), some are clearly overrepresented because of their presence in the repeats of the CRISPR sequences. For example, the overrepresented *word* ACTATAGA, included in the CRISPR repeats, is repeated 196 times (see the electronic supplementary material, figure S3).

For most of the other candidates, no obvious biological function could directly be inferred. There is evidence for eukaryotes that the non-random pattern of short words (two to four letters) may be due to evolutionary changes in informational processes such as DNA replication and repair, and the pattern of long *words* (eight letters) may reflect evolutionary changes in gene regulatory machinery. The presence of such long *words* may reflect a non-random frequency of the DNA-binding sites specific for transcription factors [39]. This observation might indicate the presence of eukaryotic-like transcriptional regulation in Archaea.

### 4.5. Structure and dynamics of the *Sulfolobus islandicus* genomes

The structural comparison of two closely related genomes, HVE10/4 and REY15A, was published in 2011 [7]. It revealed a high level of synteny of gene content for these genomes and the presence of two variable regions, one of about 0.5–0.7 Mb and a second corresponding to a 200 kb inversion. The structure of the LAL14/1 genome was compared with those of the HVE10/4 and REY15A genomes by dot-plot analyses (see the electronic supplementary material, figure S4). This revealed a well-conserved 2 Mb core region common to all three genomes, with substantial synteny conservation. Many rearrangements were detected in the variable regions of each genome. The localization, length and genetic context of all major differences detected by the dot-plot approach are listed in the electronic supplementary material, table S6. Previous analysis revealed a large inversion of 0.5 Mb as one of the major differences between HVE10/4 and REY15A genomes [7]. Genome sequencing of the LAL14/1 in combination with the phylogenetic analysis (figure 1) allowed us to infer that the inversion occurred in the ancestor of the HVE10/4, while LAL14/1 retained the ancestral genome organization.

**Figure 3.**
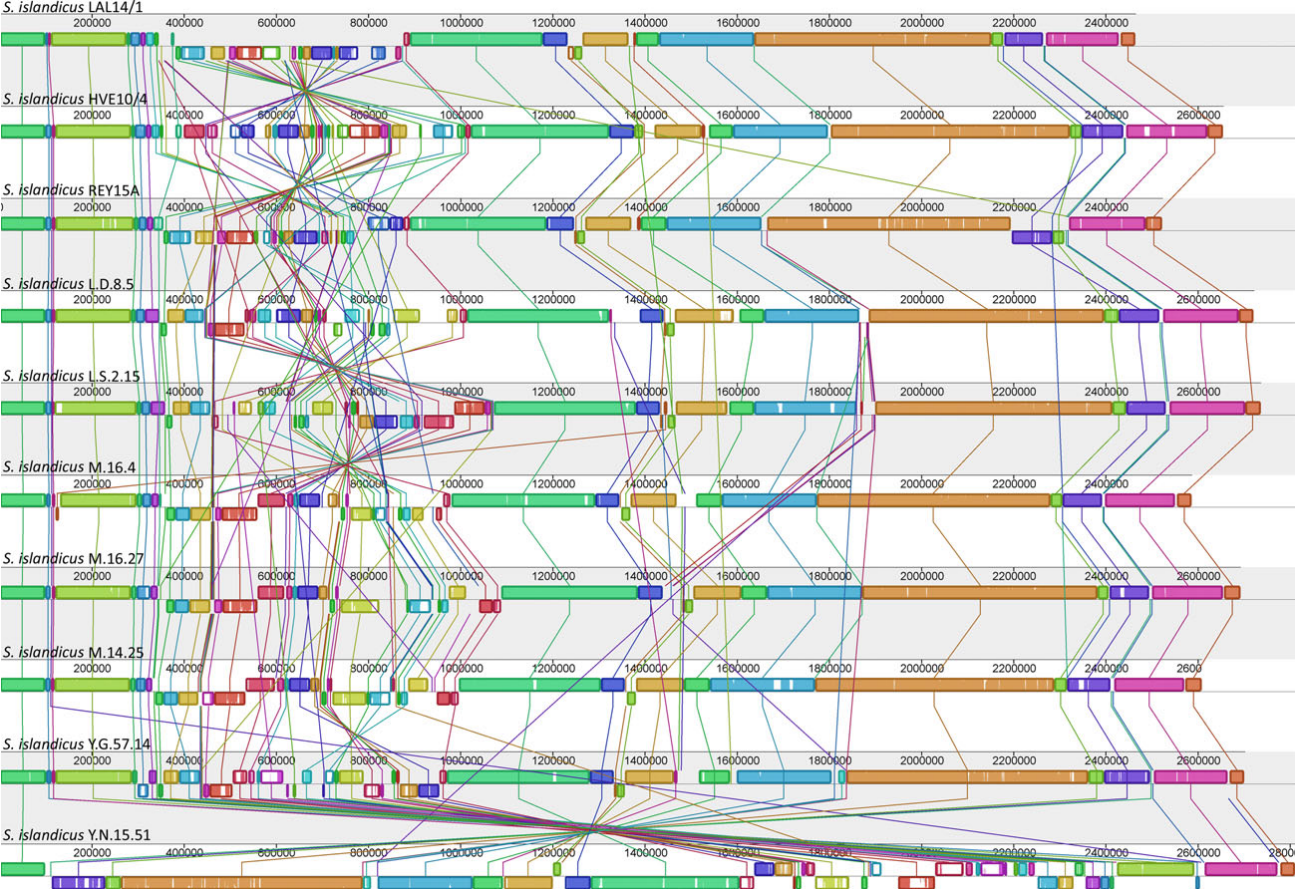
Alignment of 10 *S. islandicus* genomes by *progressiveMauve* approach*.* The blocs of the same colours indicate the regions of synteny. The unique regions are indicated by the absence of blocs.

The program *progressiveMauve* [41] was used for alignments of the 10 *S. islandicus* genomes and visualization of genome rearrangements (figure 3). All the genomes have a common general organization, with a well-preserved part covering about 75 per cent of the genome and a long variable region. Small strain-specific variable regions are scattered throughout the conserved regions of all of the 10 genomes analysed; many correspond to the insertion of heterologous genes transferred horizontally (see below).

**Figure 4.**
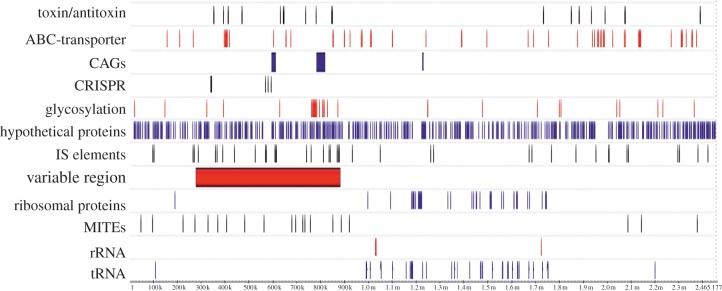
Distribution of some families of genes in the *S. islandicus* LAL14/1 genome compared with the position of the large variable region.

Each genome contains several relatively small specific regions and all of them have a unique long variable region situated in the same segment of the genome (see the electronic supplementary material, table S7). These variable regions range in size from 587 to 802 kb and have very heterogeneous genetic contexts. The largest variable region in *S. islandicus* LAL14/1 is 608 kb (between positions 282 and 890 kb) and represents 24.7 per cent of the genome. It does not contain any known essential genes, such as those for tRNA, rRNA or ribosomal proteins, or the replication origin sites (*ori*). Variable regions are usually preferential sites for integration and accumulation of non-essential genes in the genome [7] and the LAL14/1 genome is not an exception. The variable region of LAL14/1 carries most of its integrative elements, including both functional and inactivated IS, MITEs (miniature inverted-repeat transposable elements), the two largest CAG regions, all of the identified CRISPR/*cas* and *cmr* modules, half of the toxin/antitoxin genes, and many of the putative glycosylase genes (figure 4).

### 4.6. Analysis of tRNA genes, integration events and horizontal gene transfer

The pattern of tRNA genes in *S. islandicus* LAL14/1 is the same as those in *S. islandicus* REY15A and HVE10/4 [7] and very similar to those in other sequenced *S. islandicus* strains. The LAL14/1 chromosome carries 45 functional tRNA genes (see the electronic supplementary material, table S8) all located in the conserved regions. Sixteen of the tRNA genes include intron sequences (12–65 nt long) immediately downstream from the anticodon triplet. The genes for the tRNA^glu^[CTC] and tRNA^glu^[TTC] each have an insertion in their D-loop.

In Sulfolobales, the tRNA genes are preferential sites of integration of conjugative plasmids and fuselloviruses [4,42,43]. The mechanism of integration usually involves site-specific recombination between the tRNA gene target and the integrase gene (*int*) carried by an extrachromosomal element [44]. The presence of remnants of the corresponding *int* gene, overlapping the sequence of the tRNA gene target, often serves as a strong indication for an ancestral integrative event. The sequences of the remnants of the integrative elements are often incomplete or extensively degenerated, making their *in silico* identification challenging.

To identify the potential integrated extrachromosomal elements in the LAL14/1 genome, we set out to locate gene clusters enriched in homologues of proteins encoded by archaeal plasmids and viruses. For this purpose, all LAL14/1 proteins were compared (BLASTp) against the local protein database containing sequences of publicly available archaeal viruses (fifty-five) and crenarchaeal plasmids (twenty-five). Genomic loci containing at least five plasmid/viral homologues per 20 kb region were retained and manually inspected. This approach led to identification of four putative integrated elements. Notably, none of them appears to be functional, as judged from their incomplete gene complements when compared with ‘autonomous’ elements and lack of identifiable attachment sites. Three elements (SiL-E1 [SiL_0398..SiL_0402], SiL-E2 [SiL_1310..SiL_1321], SiL-E3 [SiL_1467..SiL_1481]) are likely to be remnants of conjugative plasmids, while the fourth one (SiL_2367..SiL_2371) is related to SSV-like fuselloviruses. SiL-E2, SiL-E3 and the SSV-like element are located in the proximity of different tRNA genes, which probably served as their respective integration targets, while SiL-E1 was found within the CRISPR_2 locus (see below).

In order to uncover the potentially more ancient integration events at the tRNA genes, which could have eluded identification using the criteria detailed above, we have inspected the genomic regions proximal to other LAL14/1 tRNA genes for the presence of plasmid/viral homologues and compared the obtained patterns with those of HVE10/4 and REY15A.

In general, the pattern of insertions linked to the tRNA genes in the LAL14/1 strain is similar, but not identical, to those in REY15A and HVE10/4 (table 5).

**Table 5.** Overview of integration events targeting the tRNA genes in three *S. islandicus* strains, LAL14/1 (present work) and REY15A and HVE10/4 [7]. For the corresponding lanes of the table, the sequences found in three strains are given in footnotes a–c. In the CAG column the main digit gives the number of genes and the upper small digit indicates the CAG score rated from 1 (most atypical) to 10 (not atypical). CAGs written in bold are detailed in table 6.

tRNA	intron	integration events					
		REY15A		HVE10/4		LAL14/1	
		BLASTp	CAG	BLASTp	CAG	BLASTp	CAG
Val-TAC	no	SiRe1242–1247 conj. plasmid	5^2–6^	SiH1814	1^1^	—	—
Phe-GAA^a^	no	SiRe1321–SiRe1323 conj. plasmid	9^1–7^	SiH1399–1402 conj. plasmid	4^5–7^	SiL1310–1321 Sil-E2 conj. plasmid	6^1–5^ **CAG3**
Met-CAT	yes	SiRe1465–SiRe1479	**15^1–7^** **CAG4**	SiH1557–1560	4^2–7^	SiL1467–1481 Sil-E3 conj. plasmid	13^1–5^
Glu-TTC^b^	no	intN fragment^c^ SiRe1484–1490 conj. plasmid	7^3–7^	intN fragment^c^ SiH1561–1574 conj. plasmid	12^1–7^	intN fragment SiL1484–1485 conj. plasmid	5^1–3^
Ala-GGC^a^	no	intN fragment	1^6^	intN fragment	1^7^	intN fragment	1^5^
Thr-GGT	no	SiRe2413–2417 SSV-like	3^1^	SiH2464–2472 SSV-like	**5^1 to 2^** **CAG 7**	SiL2367–2371 SSV-like	3^1^
Pro-GGG^a^	yes	intN fragment	1^3^	intN fragment	1^3^	intN fragment	1^3^
His-GTG^b^	no	SiRe1787–1792	12^1–7^	SiH1866–SiH1871	5^1–6^	—	—
Leu-TAA^a^	yes	SiRe1255; SiRe1257	2^5 and 4^	SiH1333; SiH1335	2^5 and 4^	SiL1246–SiL1248	3^2–7^
Arg-GCG	no	—	—	SiH1544 transposase	1^5^	—	—
Ser-GGA^a^	no	SiRe1778–1779	2^3 and 5^	SiH1858–1859	2^3 and 5^	SiL1773–SiL1774	2^3 and 5^
Lys-CTT^b^	yes	—	—	SiH1917 transposase	1^1^	SiL1826; SiL1828	2^2 and 4^
Ala-CGC	no	SiRe1038 hyp. protein	1^1^	—	—	—	—
Val-GAC	no	SiRe1734 hyp. protein	1^1^	—	—	—	—
Pro-TGG	no	SiRe1936–1939 transposase and hyp proteins	4^1–5^	—	—	—	—
Leu-CAG	no	—	—	—	—	—	1^1^
Gly-GCC	no	—	—	—	—	—	1^1^
Arg-CCT	yes	—	—	—	—	—	1^1^

^a^Nearly identical.

^b^Distantly related sequences.

^c^Not mentioned by [7].

Some of the insertions are clearly strain-specific (insertions into the tRNA^Ala^[CGC], tRNA^Val^[GAC], tRNA^Pro^[TGG], tRNA^Leu^[CAG], tRNA^Gly^[GCC], tRNA^Arg^[CCT] and tRNA^Arg^[GCG] genes). Other are present in two (insertions in tRNA^Lys^[CTT] and tRNA^His^[GTG]) or all three strains (tRNA^Leu^[TAA] and tRNA^Ser^[GGA] genes). In the case of 7 tRNA genes (tRNA^Phe^[GAA], tRNA^Glu^[TTC], tRNA^Ala^[GGC], tRNA^Thr^[GGT], tRNA^Pro^[GGG], tRNA^Leu^[TAA] and tRNA^Ser^[GGA]), the three strains carry nearly identical remnants of the same integrated elements. This suggests that the respective integration events occurred in the common ancestor of REY15A, HVE10/4 and LAL14/1.

Proviruses are common companions of archaeal genomes [45–47]. Thus, it was somewhat surprising not to find potentially functional proviruses in the LAL14/1 genome. The only virus-derived element of LAL14/1 integrated in the tRNA^Thr^[GGT] gene is a highly degenerated remnant of an SSV-like fusellovirus. Notably, the element does not appear to be closely related to any particular fusellovirus, since different genes display affinities to distinct fuselloviruses. To gain an insight into the timeframe of this viral integration event, we analysed the equivalent loci in all available *S. islandicus* genomes. The traces of SSV integration were found in all *S. islandicus* strains, except for the M.16.27, which contained a gene for the pNOB8-type integrase at the equivalent position [48]. Interestingly, in all cases the elements were severely degenerated; a selection of genomic alignments can be found in the electronic supplementary material, figure S5. The most parsimonious scenario for the observed distribution of SSV-like remnants in *S. islandicus* genomes involves a single event of SSV-like virus genome integration into the tRNA^Thr^[GGT] gene, followed by gradual deterioration of the provirus along the evolutionary history of *S. islandicus* species. The integration has probably occurred following the divergence of *S. islandicus* and *S. solfataricus* from their common ancestor, since *S. solfataricus* lacks a detectable SSV-like element at the equivalent genomic locus.

Identification of insertions by BLASTp analysis may be hampered by the insufficient conservation of the inserted genes or by limited coverage of the diversity of archaeal mobile genetic elements. Furthermore, some of the insertions could occur in loci other than the tRNA genes. To overcome these caveats, we have applied a BLASTp-independent approach based on the search of CAGs [17,49]. Following this approach, the putative integrated elements could be identified following their atypical codon usage compared with that of the conserved part of the host chromosome. For LAL14/1, HVE10/4 and REY15A the results obtained by this approach are summarized in table 6, and a brief general comparison of CAG distribution in 10 *S. islandicus* genomes is present in the electronic supplementary material, table S9. Notably, nearly all insertions detected in LAL14/1, HVE10/4 and REY15A by the BLASTp analyses were confirmed by the CAG approach; in addition, some of the integrative events were only predicted by the CAG search. LAL14/1 has three CAG regions (CAG1–3) of 14.6, 32.5 and 2.5 kb that carry 43 genes with atypical codon usage. CAG3 was found to correspond to SiL-E2 element integrated into tRNA^Phe^[GAA] identified by BLASTp analysis, while CAG1 and CAG2 could not be predicted by other approaches.

**Table 6.** Major CAG regions and corresponding predicted functions in *S. islandicus* LAL14/1, HVE10/4 and REY15A.

strain/CAG	position	no. of atypical genes^a^	description
*S. islandicus* HVE10/4			
CAG 1	497317–519235	15 (5)	transposase IS200/IS605
CAG2	554497–563952	8 (4)	gene *orfB*
CAG3	725905–750622	17 (4)	genes *vapBC*, CRISPR_3 of family III and six genes *cas*
CAG4-1	895122–912372	20 (6)	genes of hydrogenases and ABC transporter; HVE10/4 specific
CAG4-2	921672–938729	16 (9)	genes of hydrogenases; HVE10/4 specific
CAG5	967655–977019	11 (9)	HVE10/4 specific
CAG6	1399248–1420774	14 (7)	restriction–modification system of type I found HVE10/4 specific
CAG7	2380543–2386271	6 (6)	insertion in tRNA[Thr]^GGT^
*S. islandicus* REY15A			
CAG 1	555326–562352	7 (4)	partially similar to CAG1 of LAL14/1 and to CAG3 of HVE10/4; *vapBC*
CAG2	722610–725192	5 (4)	REY15A specific
CAG3-1	790437–804374	17 (13)	transposase IS5
CAG3-2	829169–837249	6 (4)	glycosyl transferase gene
CAG3-3	845351–852630	7 (1)	IS200/605; *vapBC*; genes *cas*
CAG4	1372809–1383519	12 (6)	insertion in tRNA[Met]^CAT^; *vapBC*
*S. islandicus* LAL14/1			
CAG1	600528–615132	10 (4)	*vapBC*; gene *csm6-like*
CAG2	789437–821915	26 (16)	genes of methyltransferases and glycosyltransferases
CAG3	1232632–1235126	7 (5)	inserts in tRNA[Phe]^GAA^

^a^The first digit indicates the total number of genes in the CAG region and the digit shown in the parenthesis indicates those for which the function could not be predicted.

Some of the functions identified as being associated with the CAG loci are: the restriction–modification system I characteristic of HVE10/4; some elements of CRISPR-based immunity in HVE10/4 and LAL14/1; and various enzyme families (methyl- and glycosyltransferases, hydrogenases). The genes transferred horizontally and integrated into the chromosomes of these *S. islandicus* strains include many transposons and toxin/antitoxin gene pairs of the *vapBC* family.

To summarize, LAL14/1 carries the remnants of 13 insertion events into the tRNA genes, and three additional elements (SiL-E1, CAG1 and CAG2) are integrated into other loci; the same or a similar number of insertions is found in HVE10/4 and REY15A. These results further illustrate the fact that tRNA genes are frequently attacked by various mobile genetic elements. The observation that all (or at least the majority) of the integrated elements appear to be non-functional suggests that LAL14/1 possesses an efficient mechanism of purging its genome of unwelcomed insertions.

### 4.7. Replication origins, *oriC*

The positions of the replication origins in the chromosome of LAL14/1 were predicted by two independent approaches, Z-curve [50,51] and ACCA-plot [52], that produced very similar results (see the electronic supplementary material, figure S6). Consistent with all other Sulfolobales genomes analysed *in silico* [7,53] or *in vivo* [54–56], three *oriC* origins of replication were detected. Their positions and genomic contexts are well conserved with respect to other *Sulfolobus* genomes (see the electronic supplementary material, table S10 and [7]). The *oriC1* site (mapped at position 1.59 Mb) is linked to the *cdc6-1* gene (SiL_0002), *oriC2* (position 800 bp) is linked to the *cdc6-3* gene (SiL_1733), and *oriC3* (position 1.15 Mb) to the *cdc6-2*/*whiP* genes (SiL_1228/SiL_1206).

**Figure 5.**
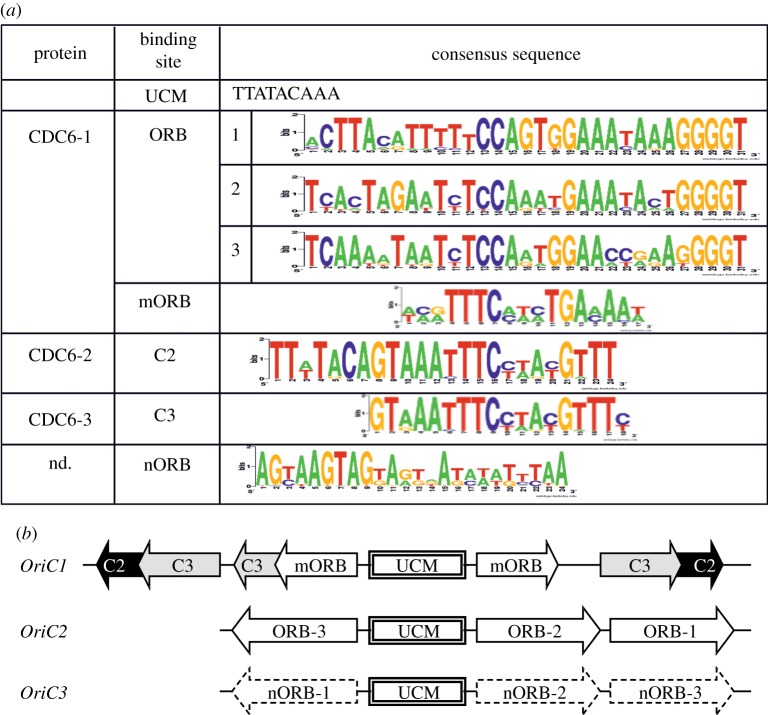
Conserved sequences and structural organization of replication origins *oriC* in *S. islandicus*. (*a*) Consensus DNA sequences present in *oriC* sites. The size of the letters is directly proportional to the residue conservation. (*b*) Structural organization of three *oriC* in *S. islandicus*.

The structures of the *oriC1* and *oriC2* sites in *S. solfataricus* P2 are well characterized [56,57], and these two replication origins are organized similarly in *S. islandicus* LAL14/1 (figure 5). Their central AT-rich UCM sequence (uncharacterized motif) is surrounded by the characteristic ORB sequences (origin recognition box). The *oriC1* site also contains additional specific palindromic sequences, called C2 and C3, that are recognized by the replication initiation proteins Cdc6-1, Cdc6-2 and Cdc6-3 [56].

In Sulfolobales, the *oriC3* site is usually linked to the *whiP* gene. A comparative analysis of *oriC3* in the 10 *S. islandicus* strains and in *S. solfataricus* P2 provided new insights into the organization of this region: we identified three conserved ORB-like sites (nORB) upstream and downstream from the typical UCM site (figure 5). The *oriC2* and *oriC3* sites are organized similarly and, unlike *oriC1*, do not contain the C sequences.

### 4.8. Toxin–antitoxin systems

A family II (VapBC) toxin–antitoxin (TA) system is present in many Archaea and is very abundant in Sulfolobales [7,58,59]. All *S. islandicus* strains carry many TA gene pairs of the VapBC family as well as genes of another family considered to play a TA role [60]: HEPN-NT (*Higher Eukaryotes and Prokaryotes Nucleotide-binding-Nucleotidyl Transferase*; electronic supplementary material, table S11).

Eight of the 15 *vapBC* gene pairs in *S. islandicus* LAL14/1 map in the variable region of the genome. The other seven *vapBC* gene pairs map in the conserved part of the genome and all share a similar genetic context in the three strains analysed. For four of these loci (SiL_2040/2041, SiL_2042/2043, SiL_2080/2081, SiL_2253/2254), the genomic context is particularly well preserved. All *vapBC* loci, except SiL_2575/2576, are flanked by degenerated copies of *IS* elements, probably involved in the transposition of *vapBC*.

As observed in HVE10/4 and REY15A [7], the *vapB* (toxin) and *vapC* (antitoxin) genes of different subtypes were found in *S. islandicus* LAL14/1 in various combinations giving different variants of the *vapBC* operon (data not shown). This combinatorial diversity of *vapBC* gene pairs may indicate the existence of several types of the toxin/antitoxin mechanisms. All *vapB* and *vapC* gene combinations found in *S. islandicus* LAL14/1 are also found in HVE10/4 and REY15A, except for SiL_0413/0414 and SiL_0631/0632 combinations present in LAL14/1 and HVE10/4 but not in REY15A.

Members of the toxin/antitoxin family HEPN-NT were detected in all *S. islandicus* strains, with multiple copies of the corresponding genes (see the electronic supplementary material, table S12). Unlike the *vapBC* system*,* HEPN-NT gene pairs are stable and each HEPN gene type is strictly associated with its specific NT gene type. HEPN-NT operons are classified into two subfamilies, I and II. Subfamily I is ubiquitous and all of its representatives in the 10 *S. islandicus* genomes analysed both occupy the same genetic regions and are always localized in conserved parts of the genomes. Subfamily II is much more diverse. Its representatives in *S. islandicus* map in both conserved and variable regions of the chromosome. Note that many copies of HEPN-HT family II pairs include only truncated forms of the HEPN gene and are not functional.

The production of sulfolobicins, a type of toxin that inhibits the growth of sensitive *Sulfolobus* strains, is characteristic of two other well-studied Sulfolobales, *S. acidocaldarius* and *S. tokodaii* [61–63]. No sulfolobicin-encoding genes, such as *sulA*, *sulB* and *sulC*, were found in any of the 10 *S. islandicus* genomes, indicating the absence of this toxin system from these species. Nevertheless, a truncated copy of the *sulA* gene, which obviously cannot code for a functional toxin, is present in *S. islandicus* REY15A [61].

### 4.9. UV-inducible type IV pili

Many Sulfolobales (ex. *S. solfataricus*, *S. tokodaii* and *S. acidocaldarius*) code for a UV-inducible type IV pilus system that promotes cellular aggregation and efficient exchange of chromosomal markers [64,65]. The formation of pili is controlled by the UV-inducible *ups* operon which comprises five genes: *upsX, upsE, upsF, upsA* and *upsB* [4–6]. This operon is present in all 10 *S. islandicus* strains (and all other sequenced species of Sulfolobales, electronic supplementary material, table S13) and is in all cases in the conserved part of the genome. The Ups proteins encoded by all *S. islandicus* are very similar and form a specific phylogenetic group within the Ups family in Sulfolobales (see the electronic supplementary material, figure S7).

The *in silico* data strongly suggest that the *ups* locus of *S. islandicus* is functional *in vivo*, and it very probably plays the same biological role as in *S. solfataricus*, *S. tokodaii* and *S. acidocaldarius*.

### 4.10. Insertion sequence elements and miniature inverted-repeat transposable elements

*Sulfolobus islandicus* LAL14/1, as HVE10/4 and REY15A, contains several families of *IS* elements with members present in multiple copies [7] (see the electronic supplementary material, table S14).

The *orfB*-containing *IS* (families *IS*605 and *IS*200/605) considered to be ancestral for the archaeal domain [66,67] are overrepresented in all three *S. islandicus* strains analysed: 95% of the copies of the *orfB* gene not linked to *orfA* in HVE10/4, REY15 and LAL14/1 were predicted to be functional.

Only seven of the 53 structurally valid *IS* (*IS* with intact inverted terminal repeats (ITRs)) in the LAL14/1 genome are predicted to code for functional transposases; transposase genes in the remaining 46 *IS* are truncated. The *IS* patterns of the two other strains, HVE10/4 and REY15A, are very different. Most of the *IS* in these genomes (45/76 in HVE10/4 and 52/96 in REY15A) code for a full-length transposase and are therefore predicted to be functional. However, many of the mutated *IS* may be mobilized by transposases of the same family encoded in *trans* [68]. Thus, 62 of the 76 *IS* (81.6%) detected in HVE10/4 and 87 of the 96 *IS* (90.6%) in REY15A could, in theory, be mobile (see the electronic supplementary material, table S14); the proportion is lower for LAL14/1, for which 31 of the 53 *IS* (58.4%) are potentially active.

This may indicate greater genetic stability of *S. islandicus* LAL14/1 than of either HVE10/4 or REY15A. Were this the case, strain LAL14/1 would be the most attractive model for genetic manipulations.

Another group of mobile elements in *S. islandicus* is MITEs, believed to correspond to truncated derivatives of autonomous DNA transposons [69–73]. MITEs exhibit the structural features of DNA transposons, containing terminal inverted repeats flanked by small direct repeats. The internal sequences of MITEs are short and devoid of ORFs. As non-autonomous elements, the transposition of MITEs is totally dependent on trans-acting transposases [68,74,75]. Only two classes of MITEs, SMN1 (320 bp) and SM3A (164 bp), were detected in the 10 *S. islandicus* genomes (see the electronic supplementary material, table S15). The SM3A family is more numerous in LAL14/1 than any of the other *S. islandicus* strains. The three *S. islandicus* strains from Iceland share two identical SM3A, but LAL14/1 also carries nine extra copies of SM3A that share only 95% similarity with other two SM3A copies. SMN1 transposition is dependent on the presence of a functional ISC1733 transposase and SM3A transposition on ISC1058 [68,76,77]. The MITEs of the SMN1 type in LAL14/1, HVE10/4 and REY15A could be mobilized by the ISC1733 type transposase [76] predicted to be functional in these strains. The observation of the mobilization of SMN1 in *S. islandicus* REN1H1 is consistent with this prediction [76]. None of the three *S. islandicus* strains analysed codes for a functional ISC1058 transposase, suggesting that SM3A, although present, cannot transpose in HVE10/4, REY15A and LAL14/1.

### 4.11. CRISPRs: structure, targets and phenotype

**Figure 6.**
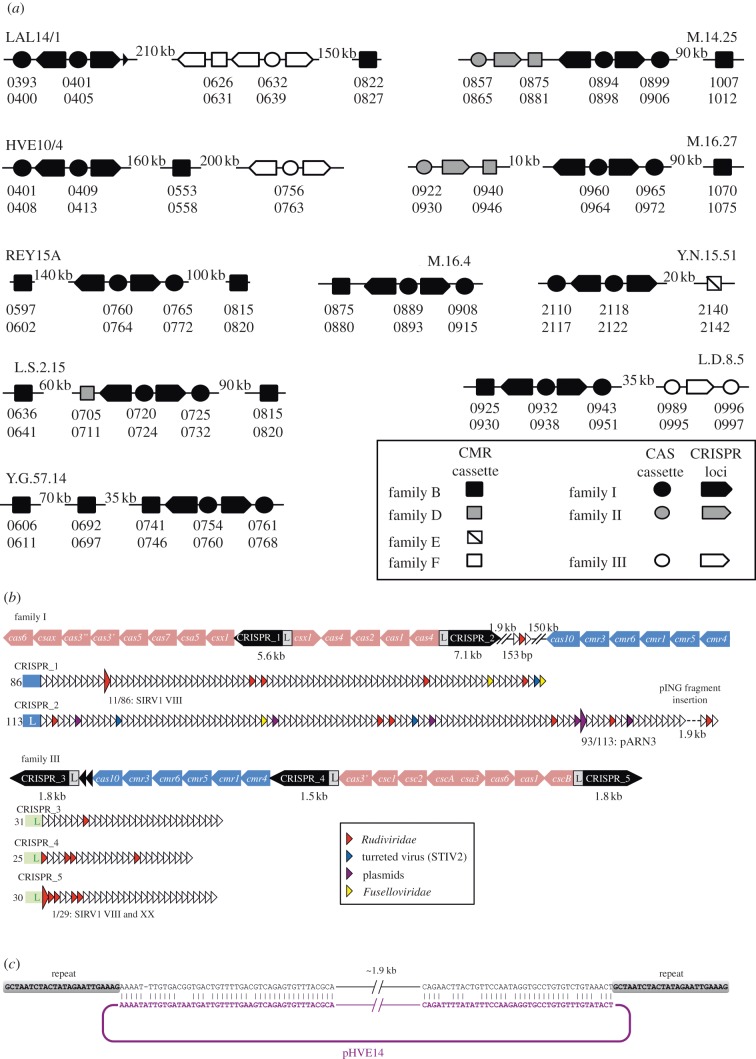
CRISPR organization in *S. islandicus* LAL14/1. (*a*) Comparison of the CRISPR structures and composition in 10 *S. islandicus* strains. (*b*) Structure and composition of CRISPRs in *S. islandicus* LAL14/1. Large-scale presentation: the orientation, position and size of CRISPR arrays (black arrows) as well as *cas* (red arrows) and *cmr* (blue arrows) gene modules are indicated. Detailed presentation: all perfectly matching spacers (large arrows) as well as selected imperfect spacers are indicated by small coloured arrows specified in the legend included in the figure body. (*c*) The pING1-like insertion in the CRISPR_2 locus is interspersed precisely between two complete 24 bp repeats, resembling by its position typical CRISPR spacers.

All sequences and genes related to the CRISPR system present in *S. islandicus* LAL14/1 (CRISPR arrays, *cas* and *cmr* gene cassettes) map within the large variable genomic region. It carries five CRISPR loci, three *cas* gene cassettes associated with subtype I-A and two *cmr* gene cassettes associated with subtype III-B [78,79]. Following the leader and repeat sequence compositions, the five CRISPRs of *S. islandicus* LAL14/1 could be divided into two families, I and III [36], while the two *cmr* modules belong to families B and F ([37]; figure 6 and table 7).

**Table 7.** Composition of CRISPRs in *S. islandicus* LAL14/1 and their putative targets. SIRV1 VIII and SIRV1 XX are different subtypes of the virus SIRV1 [34].

CRISPRs and their families	repetition	position and direction	no. of spacers	spacers with 100% identity to the indicated putative targets
*CRISPR_1* I	GCTAATCTACTATAGAATTGAAAG	344177..349768 ←L	86	11/86 SIRV1 VIII
*CRISPR*_2 I	GCTAATCTACTATAGAATTGAAAG	353820..363037 L→	113	93/113 pARN3
*CRISPR_3* III	GTAACAACACAAAGAAACTAAAAC	573686..575526 ←L	31	—
*CRISPR_4* III	GTAACAACACAAAGAAACTAAAAC	584662..586213 ←L	25	—
*CRISPR_5* III	GTAACAACACAAAGAAACTAAAAC	597345..599148 L→	30	1/29 SIRV1 VIII and SIRV1 XX

The family I CRISPR locus comprises two oppositely oriented blocks of repeat-spacer arrays separated by the first module of Cas genes. The second module is situated at the end of one of the repeat-spacer arrays (figure 6*a*). The additional Cmr module is not linked to this part and maps several hundred kilobases downstream in the genome. The family III CRISPR (figure 6*a*) comprises three clusters of spacers/repeats associated with two gene modules, one including the *cas* gene and another *cmr*. The *cmr* gene order in this module is the same as that in the Cmr module associated with the family I CRISPR.

The analysis of 285 spacers forming the CRISPR array of LAL14/1 revealed the presence of a surprisingly high number of spacers that perfectly (in three cases) or imperfectly (30 cases; electronic supplementary material, table S16) match the genomes of rudiviruses, fuselloviruses and conjugative plasmids previously described in the Icelandic hot spring environments [34,35]. The two perfectly matching spacers carried by CRISPR_1 and CRISPR_5 target the genome of the rudivirus SIRV1 [80]. Interestingly, the spacer in CRISPR_1 matches a SIRV1 gene encoding a protein, P98, responsible for formation of pyramidal structures involved in virion egress [11–13]. No spacers with 100 per cent identity to a closely related virus, SIRV2, were detected. The third perfectly matching spacer is identical to a sequence in the conjugative plasmid pARN3 [81]. Unexpectedly, we have identified an about 2 kb insertion, SiL-E1, in the CRISPR_2 locus. SiL-E1 resembles the typical CRISPR spacers in that it is interspersed between two identical repeats and is followed by additional spacer-repeat units (figure 6). This pseudo-spacer encompasses five ORFs and displays high sequence similarity to and collinearity with pING1-like conjugative plasmids of *S. islandicus* [81–83]. More specifically, SiL-E1 shares overall 82 per cent identity with plasmids pING1 [83] and pHVE14 [81]. SiL-E1 is not present in other *S. islandicus* strains. Notably, sequence similarity between SiL-E1 and pING1-like plasmids extends throughout the length of SiL-E1, leaving no unaccounted positions between the inserted sequence and the repeat regions (figure 6*c*). This suggests that SiL-E1 is unlikely to be a result of illegitimate recombination.

Some of the spacers imperfectly match DNA regions present in the genomes of other *S. islandicus* strains (M.14.25, M.16.4, Y.N.15.51, Y.G.57.14, L.D.8.5, L.S.2.15) and in *S. solfataricus* 98/2 and P2. The functions of the corresponding genes are unknown, and even the biological significance of this observation is unclear. Possibly, the genomic loci matched by these spacers represent the remnants of unknown viruses or plasmids integrated into the corresponding genomes.

The analysis of the protospacer corresponding to the spacers listed in the electronic supplementary material, table S15 allows the identification of the PAM sequence (protospacer adjacent motif). These sequences situated at the proximity of protospacers are crucial for two essential steps of CRISPR-based immunity: adaptation [84] and interference [37,85,86]. They also play an important role in the mechanism of target discrimination that prevents the recognition of chromosomal spacers as valid targets [87].

**Figure 7.**
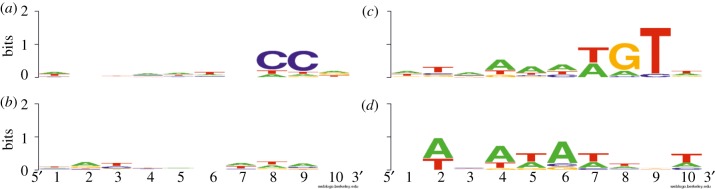
Conserved motifs in the protospacer sequences for CRISPRs I ((*a*) 5′ and (*b*) 3′) and CRISPRs III ((*c*) 5′ and (*d*) 3′).

For the LAL14/1 CRISPRs of the family I, we found the same PAM motif, **CC**, in the position (−3, −2) at the 5′ end as was already described by Gudbergsdottir *et al*. [88] (figure 7*a*). No specific PAM motif was detected at the 3’ end of these protospacers (figure 7*b*). Little is known about the protospacers corresponding to the CRISPRs of the family III. Our data indicate the existence of a conserved motif [T/A]GT occupying the position (−4, −3, −2) at the 5′ end of the protospacer (figure 7*c*). The 3′ end of these protospacers is very rich in A/T nucleotides (figure 7*d*).

### 4.12. Development of a genetic model

*Sulfolobus islandicus* LAL14/1 is a promising model for studying virus–host interaction in Archaea. LAL14/1 cells can be infected by SIRV2, a model rod-shaped virus [89] that codes for a unique mechanism of virion release: pyramidal structures form on the host cell surface, breaking the S-layer and allowing the virions to escape from the cells [11–13,90]. Investigations on SIRV2 cycle regulation and SIRV2–host interaction will require genetic tools for strain LAL14/1 as no available genetic models of *Sulfolobus* can be infected by SIRV2.

One of the most commonly used genetic markers in Archaea is the *pyrEF* operon. Pyrimidine prototrophs (Pyr+) can be easily selected, on minimal medium without uracil, after transformation of a *pyrEF*–host strain by a plasmid or viral vector carrying the wild-type *pyrEF* operon [19,91–94]. A non-reversing spontaneous *pyrEF-*deletion mutant of *S. islandicus* REY15A is widely used as a host for genetic manipulations [20]. No such mutant of *S. islandicus* LAL14/1 was available, so we constructed a *ΔpyrEF* mutant via allelic replacement approach.

To generate a *pyrEF* disruption mutant, a knockout cassette containing the *ΔpyrEF* allele from *S. islandicus* REY15A, strain E233S [19] and 1 kb regions situated downstream and upstream from *pyrEF* was obtained by PCR amplification (see §3). The *S. islandicus* LAL14/1 cells were transformed by this linear DNA fragment of 2233 bp and the *ΔpyrEF* mutants resulting from the replacement of the wild-type copy of the *pyrEF* operon on the host chromosome by a double cross-over were selected on 5′FOA (5′-fluoroorotic acid). Twenty transformants were selected and the *pyrEF* operon was analysed by PCR and sequencing; 15 of the analysed colonies carried the expected *ΔpyrEF* deletion. This mutant strain, called *S. islandicus* LAL14/1*-*CD, showed the same virus resistance/sensitivity phenotype as the parental strain (data not shown). We confirmed that this mutant is indeed derived from strain LAL14/1 by sequencing the gene coding for the A subunit of the cytochrome b558/566 (Sil_2350; the sequence of this gene is not identical in REY15A, HV10/4 and LAL14/1).

*Sulfolobus islandicus* LAL14/1*-*CD could be efficiently transformed with the pHZ2 (pRN2 replicon) [19] autonomously replicating in *S. islandicus* and carrying a wild-type copy of the *pyrEF* operon. The transformation efficiency was 10^2^–10^3^ colonies/μg of DNA.

A powerful genetic *pop-in/pop-out* approach was previously developed for another genetic model, *S. islandicus* REY15A [19]. It allows rapid and efficient creation of knockout mutants. To show that this approach is efficient in LAL14/1, we have chosen to delete one of the CRISPR loci (CRISPR_1), because CRISPR-coded functions are usually not essential for the cells in the absence of viruses and their deletion mutants are expected to be viable. Also, one of the spacers of the CRISPR_1 matches perfectly the SIRV1 virus for which LAL14/1 is resistant. If the resistance is linked to the CRISPR activity, its inactivation could decrease the level of resistance giving a detectable phenotype to this mutant.

**Figure 8.**
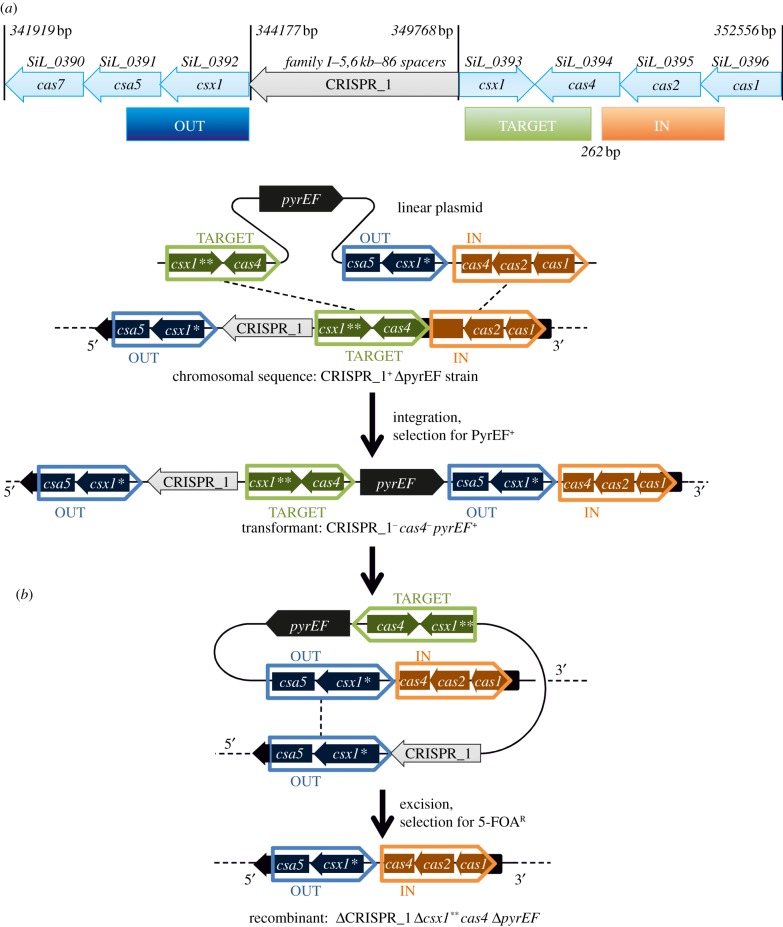
Genetic map of the CRISPR_1 region deleted by the pop-in/pop-out approach. (*a*) Genetic map of the CRISPR_1 region. The deleted region is situated between the regions OUT and TARGET. (*b*) A scheme representing two stages of recombination events generating the mutant ΔCRISPR_1Δcsx1**Δcas4Δ *pyrEF.* Two paralogues of *csx1* are present in this region. The gene csx1* corresponds to the gene Sil_0392 and csx1** to Sil_0393.

The recombinant plasmid used to inactivate the CRISPR_1 and the positions of the regions IN (867 bp), OUT (919 bp) and TARGET (776 bp) in the vector pSEF described by Deng *et al*. [19] are indicated in figure 8.

Two successive rounds of recombination (figure 8) deleted the chromosomal fragment situated between the IN and OUT regions producing a *pyrEF*+ derivative from which the CRISPR_1/*cas* region has been deleted (*ΔCRISPR_1*/*Δcsx1Δcas4*; *csx1* is annotated as Sil_0393). The deletion was confirmed by PCR analysis (data not shown). Interestingly, *ΔCRISPR_1*/*Δcsx1Δcas4* was as resistant to infection by SIRV1 as *S. islandicus* LAL14/1*-*CD. The presence of a second spacer and functional CRISPR_3 may explain this result.

The observed efficient transformation of LAL14/1 as well the ease of creation and selection of its deletion mutants indicate that LAL14/1 represents an excellent genetic model.

## 5. Discussion

*Sulfolobus islandicus* LAL14/1 is a promising model for studies on virus–host interaction and CRISPR/*cas*-based acquired immunity in hyperthermophilic Archaea. We report an extensive comparative *in silico* analysis of its genome and established this strain as genetic model.

The genome of LAL14/1 is the 10th of the species *S. islandicus* to be sequenced [9], and the third of an *S. islandicus* strain isolated in Iceland [8]. Strain LAL14/1 has the smallest known *S. islandicus* genome: it has only 2601 genes carried by a 2.47 Mb chromosome. With other sequenced *S. islandicus* strains LAL14/1 shares the same major groups of paralogous genes, most of which are transposases of various families (table 4). Its genome also encodes a large number of diverse ABC transporters, including the oligopeptide transporter. This is consistent with the high frequency of isolation of *S. islandicus* strains from enrichment cultures using rich organic media [7]. Indeed, *S. islandicus* LAL14/1 grows heterotrophically on standard laboratory liquid media with yeast extract as main carbon source.

The *S. islandicus* pan-genome contains 570 singletons representing a strain-specific set of proteins; 65 are only found in *S. islandicus* LAL14/1. As has been widely documented for other prokaryotic virus/host models (see review [95]), it is possible that some of the particular features of LAL14/1, for example, virus–host range, could be linked to the presence of specific genes or gene repertoires absent from other *S. islandicus* strains. A large proportion of the strain-specific genes map in a large variable region. In each of the *S. islandicus* genomes analysed this region covers more than 25 per cent of the chromosome length, and contains most of the transposons promoting the horizontal gene transfer and all CRISPR sequences (figure 4).

Our *in silico* analysis of the relics of mobile elements in the genomes of the three closely related *S. islandicus* strains confirms that mobile genetic elements preferentially integrate into tRNA genes. Nevertheless, searches for CAG revealed several previously undescribed long DNA segments of heterologous origin that were located in loci other than tRNA genes [49]. These clusters, most probably the consequences of genetic transfer via conjugative plasmids or other mobile genetic elements, contain 1.6 per cent of the genes in LAL14/1. Biological functions can be predicted for only a small fraction of these genes. For example, the CAGs carry several copies of *vapBC* genes of toxin/antitoxin systems, some CRISPR-related genes and genes coding for methyl- and glycosyltransferases.

A comparative analysis of the genome structure and composition of three closely related strains (HVE10/4, REY15A and LAL14/1) confirms that they have a very similar genomic pattern with a strong conservation of synteny. Nevertheless, the presence in each of these genomes of multiple local rearrangements raised the issue of the stability of the LAL14/1 genome. All three strains carry many copies of *IS* elements of various families. The transposition of an *IS* or transposon is an important source of genome instability and rearrangements in any cell [96]. Such instability is well documented in the case of REY15A, for which a relatively high incidence of the *pyrEF*-deletion mutants (one from 50 analysed PyrEF-colonies) is observed [7,19].

*Sulfolobus islandicus* LAL14/1 seems to be genetically more stable as no deletions in the *pyrEF* locus were detected by PCR analysis of 100 colonies of spontaneous LAL14/1 *pyrEF* mutants resistant to FOA (C. Jaubert, C. Danioux, G. Sezonov 2013, unpublished data). This could be due to a lower transposition activity in LAL14/1 and consequently lower frequency of genome rearrangements.

Thus, *in vivo* and *in silico* indications concerning the stability of the genome of strain LAL14/1 suggest that it would be a useful model for genetic studies. Strain LAL14/1 is the host of the model rudivirus SIRV2 [10,89,97] and has been used to study virus–host interactions in Archaea [11–13]. The availability of the sequence of the LAL14/1 genome makes global genomic analysis of the interaction between viral and host genomes during the infection cycle possible. A genetic approach would facilitate investigations of the role of particular host genes involved in this interaction, as well as the host immune response dependent on the activity of CRISPRs. We successfully inactivated two genetic loci in LAL14/1 of different sizes, *pyrEF* (2.2 kb) and CRISPR_1/*cas* (6.6 kb), using allelic replacement and in-frame markerless genetic exchange approaches. We thereby demonstrated the potential of LAL14/1 for genetic experimentation.

More than 90 per cent of the archaeal genomes analysed code for an adaptive immunity system called the CRISPR/*cas* system [98–100]. In LAL14/1, CRISPRs are represented by five loci associated with three *cas* (subtype I-A) and two *cmr* (subtype III-B) gene cassettes. The presence in the CRISPR arrays of LAL14/1 spacers matching extrachromosomal elements (SIRV1 virus, pARN1) make this strain an interesting model for studying the biological and functional role of CRISPRs in Archaea. The presence of two spacers perfectly matching the genome of SIRV1 virus allows speculation about a connexion between the CRISPR composition and the SIRV1-resistance phenotype of *S. islandicus* LAL14/1. Deletion of the CRISPR_1 carrying one of two SIRV1-specific spacers did not change the phenotype of the obtained mutant; it remained as resistant to SIRV1 as the initial strain. A construction of a double mutant *Δ*CRISPR_1 *Δ*CRISPR_5 will help to better characterize the eventual involvement of CRISPR generated immunity in resistance of LAL14/1 to SIRV1.

Recent publications report spacer acquisition under laboratory conditions in several bacterial [84,101–104] and archaeal (*S. solfataricus*) [105] models. However, analysis of the CRISPR content of 12 independent SIRV2-resistant mutants of LAL14/1 did not detect any new insertions in the CRISPR sequences (data not shown). Consequently, the acquired resistance does not appear to be related to CRISPRs and presumably involves a different mechanism of resistance [106].

LAL14/1 also carries a unique pseudo-spacer, SiL-E1, which represents an insertion of an approximately 2 kb region from a pING1-like plasmid into the CRISPR_2 array. To our knowledge, such large spacers have not been previously described in archaeal or bacterial CRISPR loci. The acquisition mechanism of this pseudo-spacer as well as its role in LAL14/1 immunity against conjugative plasmids is unclear. However, the fact that SiL-E1 is flanked by perfect repeats of CRISPR_2 (figure 6*c*) argues against the possibility of a random integration event. Plausible acquisition scenarios include faulty protospacer processing by the Cas machinery or homologous recombination between the episomal plasmid and the pre-existing CRISPR_2 spacer(s) matching the plasmid. Future studies should provide important additional information regarding spacer acquisition mechanisms and reveal whether such atypical spacers are competent in conferring immunity against mobile genetic elements in Archaea.

Physical isolation of the geothermal hot spring in which *S. islandicus* thrives makes this species a very valuable model to study microbial speciation and evolution [107]. Such studies were recently conducted on *S. islandicus* strains isolated from the hot spring located in Russia (‘M’) and USA (‘Y/N’) [9,33], and have already provided important insights into the population dynamics of hyperthermophilic Archaea. Our in-depth comparative genomics analysis clearly indicates divergence of the ‘Icelandic trinity’—LAL14/1, HVE10/4 and REY15A—from the groups ‘M’ and ‘L/Y’, supporting previously suggested biogeographical patterns of differentiation of *S. islandicus* species. Genetic tools developed in this study and those available for REY15A will help to experimentally tackle questions regarding the evolution and divergence of these Icelandic strains and compare the elucidated patterns with those available for *S. islandicus* strains isolated from other continents.

## 6. Acknowledgements

This work was supported by PhD fellowships from the ‘Ministère de l'enseignement supérieur et de la recherche’ (C.J. and C.D.) and by Pasteur-Weizmann (C.J.) allocations. We thank Nuno Peixeiro and Sophie Schbath for helpful discussions.

## Supplementary Material

Supplementary tables and figures

## Supplementary Material

Supplementary Table S2

## Supplementary Material

Supplementary Table S5
