# Supplementary material for: Genomics and genetics of Sulfolobus islandicus LAL14/1, a model hyperthermophilic archaeon
Source: Open Biol. 2013 Apr;3(4):130010. doi: 10.1098/rsob.130010 (PMC3718332; doi:10.1098/rsob.130010)
Supplement: Supplementary tables and figures [file rsob130010-s1.doc]

**C. Jaubert et al., Genomics and genetics of *Sulfolobus islandicus* LAL14/1, a model hyperthermophilic archaeon**

**Supplementary materials**

| **Nodes families** | **Present in *S. islandicus***  **pan-genome** | **Present in *S. islandicus* LAL14/1** |
| --- | --- | --- |
| *IS*H3 transposases, ISC1200 | 142 | 3 |
| *IS*110 transposases, ISC1229 | 113 | 2 |
| CoA pathway genes | 68 | 5 |
| ATPase subunit of the ABC transporters | 63 | 5 |
| *IS*1 transposases, ISC796 | 53 | 9 |
| *IS*607 transposases, ISC1921 | 48 | 0 |
| *IS*6 transposases, ISSis1 and ISSto2 | 48 | 2 |
| Putative molybdopterins | 40 | 4 |

**Table S1.**Functions associated to the most represented *nodes* in the *S. islandicus* pan-genome and their representatives in *S. islandicus* LAL14/1.

| Locus_tag | Predicted function |
| --- | --- |
| **SiL_0127** | Hypothetical Protein |
| **SiL_0155** | Hypothetical Protein |
| **SiL_0303** | Hypothetical Protein |
| **SiL_0329** | Hypothetical Protein |
| **SiL_0384** | Hypothetical Protein |
| **SiL_0409** | Hypothetical Protein |
| **SiL_0481** | Putative Acyl-coenzyme A synthetases/AMP-(fatty) acid ligases |
| **SiL_0498** | Hypothetical Protein |
| **SiL_0587** | Putative methyltransferase small |
| **SiL_0600** | CRISPR-associated protein, Cmr3 |
| **SiL_0601** | CRISPR-associated protein, Cas10 |
| **SiL_0603** | CRISPR-associated protein, Cmr5 |
| **SiL_0604** | CRISPR-associated protein, Cmr1 |
| **SiL_0614** | Hypothetical Protein |
| **SiL_0630** | Related to a CRISPR-associated protein |
| **SiL_0657** | Hypothetical Protein |
| **SiL_0686** | Hypothetical Protein |
| **SiL_0706** | Hypothetical Protein |
| **SiL_0762** | Hypothetical Protein |
| **SiL_0794** | Hypothetical Protein |
| **SiL_0799** | Hypothetical Protein |
| **SiL_0810** | Hypothetical Protein |
| **SiL_0818** | Putative Glycosyltransferase |
| **SiL_0823** | Hypothetical Protein |
| **SiL_0837** | Hypothetical Protein |
| **SiL_0838** | Hypothetical Protein; distantly related to ATV_gp71 |
| **SiL_0839** | Membrane protein involved in the export of O-antigen and teichoic acid |
| **SiL_0855** | Hypothetical Protein |
| **SiL_0857** | Hypothetical Protein |
| **SiL_0860** | Hypothetical Protein |
| **SiL_0874** | Hypothetical Protein |
| **SiL_0876** | Hypothetical Protein |
| **SiL_0890** | Hypothetical Protein |
| **SiL_0909** | Hypothetical Protein |
| **SiL_1006** | Hypothetical Protein |
| **SiL_1086** | Hypothetical Protein |
| **SiL_1316** | Hypothetical Protein |
| **SiL_1318** | Hypothetical Protein |
| **SiL_1322** | Hypothetical Protein |
| **SiL_1325** | Hypothetical Protein |
| **SiL_1330** | Hypothetical Protein |
| **SiL_1477** | Hypothetical Protein |
| **SiL_1655** | Hypothetical Protein |
| **SiL_1730** | Hypothetical Protein |
| **SiL_1761** | Hypothetical Protein |
| **SiL_1951** | Hypothetical Protein |
| **SiL_1973** | Hypothetical Protein |
| **SiL_2104** | Hypothetical Protein |
| **SiL_2115** | Hypothetical Protein |
| **SiL_2365** | Hypothetical Protein |
| **SiL_2366** | Hypothetical Protein |

A

| Locus_tag | Predicted function |
| --- | --- |
| **SiL_0408** | Hypothetical protein |
| **SiL_0507** | Hypothetical protein |
| **SiL_1048** | Hypothetical protein |
| **SiL_1321** | Conjugative plasmid protein |
| **SiL_1473** | Fe-S oxidoreductase |
| **SiL_1752** | Hypothetical protein |
| **SiL_1913** | Hypothetical protein |

B

| Locus_tag | Predicted function |
| --- | --- |
| **SiL_0405** | Putative MarR family transcriptional regulator |
| **SiL_0602** | CRISPR-associated protein Cmr6 |
| **SiL_0605** | CRISPR-associated protein Cmr4 |
| **SiL_1009** | Hypothetical protein |
| **SiL_1319** | Predicted secreted endonuclease |
| **SiL_1320** | Conjugative plasmid protein |
| **SiL_2368** | Viral protein (SSV-like) |

C

**Table S3. List of the genes of *S. islandicus* LAL14/1 specific to (A) *S. islandicus* LAL14/1 (B) Sulfolobus; (C) *Crenarchaeota*.**

| **Word**  **length** | **Total number of distinct words** | **Most overrepresented words** | **Most underrepresented words** |
| --- | --- | --- | --- |
| **2** | 16 | ag/ct; cc/gg | ac/gt; *cg* |
| **3** | 64 | acg/cgt; cca/tcc; gga/tgg | aca/tgt; cga/tcg; *gac/gtc* |
| **4** | 256 | cagc/gctg; catc/gatg; cctc/gagg; gaaa/tttc | attc/gaat; cagg/cctg; gaac/gttc; gagc/gctc; |
| **5** | 1024 | aatcc/ggatt; aattc; catta; taagg; tgatg | aatca; agtaa; attca; catcc; gaaac; ggatg; gtttc; tgaat; tgatt; ttact |
| **6** | 4096 | aaatta; **ctacta**; cttaac; cttagc; gcagga; gctaag; gttaag; taaaaa; taattt; tagtag; tcctgc; ttttta | aaaaaa/tttttt; aagtag; *ggtacc*; *gttaac*; gttatc; *taatta* |
| **7** | 16384 | atttttt; caacttc; cattttc; gaaatag; gaatttg; gatactg; gattacg | aaaaaaa/ttttttt; catatta; cattcta; taaatag; tagattg; taggatg; taggttg |
| **8** | 65536 | **actataga/tctatagt**; attgaaag/ctttcaat; **ctaatcta**; ctttgtgt; **taatctac**; **tagaattg**; **tctactat**; **ttcaattc** | aaatctac; aatagaat; *actatagt*; cttgtacc; gattttac; tagattaa/ttaatcta; *tctataga*; ttgttgtt |

**Table S4. Most under- and overrepresented words in the genome of *S. islandicus* LAL14/1.**

Some of the most exceptional words are shown and were selected according to their R’MES rank. *Palindromic words* are indicated as underlined words in italic characters. Words of length 6 to 8 related to CRISPR repeats are indicated in bold characters. The words are grouped with their reverse complement (word/ reverse complement) whenever relevant.

| Dot-plot LAL14/1 vs HVE10/4 | | |
| --- | --- | --- |
| Position | | Description |
| LAL14/1 | HVE 10/4 |  |
| 344.209 bp -  381.042 bp  (36,8kb) | - | Absent in HVE 10/4.  LAL14/1 – contains CRISPRs family I |
| - | 392.309 bp -  452.067 bp (59,8kb) | Absent in LAL 14/1 |
| 381.042 bp -  871.850 bp (490,8kb) | 452.067 bp -  1.014.813 bp (562,7kb) | Inversion. Variable region. Presence of CRISPRs of family III in LAL14/1. |
| 766.321 bp -  833.855 bp (67,5kb) | - | Absent in HVE 10/4.  Contains 73ORFs including methyltransferase (20 ORFs) and glycosylase (11 ORFs) genes |
| 871.850 bp -  344.209 bp (1.957kb) | 1.014.813 bp -  392.309 bp  (2.032kb) | Conserved in both strains. |
| 1.230.660 bp -  1.265.311 bp (34,7kb) | 1.374.116 bp -  1.421.105 bp (47kb) | Inversion. Contains 3 ARNt genes and 43 ORF of unknown function |

**A**

| Dot-plot LAL14/1 vs REY15A | | |
| --- | --- | --- |
| Position | | Description |
| LAL 14/1 | REY15A |  |
| 317.849 bp -  353.843 bp (36kb) | 721.233 bp -  768.241 bp  (47,1kb) | Inversion. Variable region. LAL14/1 -presence of CRISPRs family I |
| 353.843 bp -374.796 bp (21kb) | - | Absent in REY15A. |
| 374.796 bp -  736.187 bp  (361,3kb) | 333.277 bp -  721.233 bp  (388kb) | Conserved in both strains. Variable region |
| 736.187 bp -842.637 bp (106,5kb) | 768.241 bp -874.957 bp (106,7kb) | Inversion. Variable region.. |
| 762.101 bp -806.736 bp (44,6kb) | - | Absent in REY15A. |
| 842.637 bp -875.499 bp (32,8kb) | - | Absent in REY15A. Contains genes *vapBC* and some genes coning for the ABC transporters. |
| 875.499 bp -  317.849 bp  (1.907,5kb) | 874.957 bp -  333.277 bp  (1.981,3kb) | Conserved in both strains. Well concerved in all *S. islandicus.* |
| 2.153.028 bp -  2.264.012bp  (111kb) | 2.189.662bp -  2.313.104bp  (123.4kb) | Inversion |

**B**

**Table S6. Comparison of genome structures by dot-plot analysis.** (A) *S. islandicus* LAL14/1 *vs* HVE 10/4. (B) *S. islandicus* LAL14/1 *vs* REY15A.

| ***S. islandicus* strain** | **Position in the genome from..to..** | **Variable region size in kb** |
| --- | --- | --- |
| LAL 14/1 | 282-890 | 608 |
| HVE 10/4 | 294-1019 | 725 |
| REY 15A | 304-891 | 587 |
| LD8.5 | 294-1013 | 719 |
| LS2.15 | 310-1078 | 768 |
| M14.25 | 302-1001 | 699 |
| M16.27 | 296-1090 | 794 |
| M16.4 | 310-980 | 670 |
| YG57.14 | 300-973 | 673 |
| YN15.51 | 2407-1605 | 802 |

**Table S7. Positions and sizes of variable regions in different strains of *S. islandicus***

| **Position** | **tRNA type**  **Anti-codon** | | **Intron start** | **End** | **Intron** |
| --- | --- | --- | --- | --- | --- |
| 994191..994264 | Ala | CGC | - | - | No |
| 1484693..1484766 | Ala | GGC | - | - | No |
| 1594052..1594125 | Ala | TGC | - | - | No |
| 1570342..1570416 | Arg | CCG | - | - | No |
| 1526901..1526988 | Arg | CCT | 1526940 | 1526952 | Yes |
| 1353142..1353216 | Arg | GCG | - | - | No |
| 1161877..1161951 | Arg | TCG | - | - | No |
| 1568617..1568706 | Arg | TCT | 1568656 | 1568670 | Yes |
| 1759525..1759611 | Asn | GTT | 1759574 | 1759561 | Yes |
| 1633028..1633100 | Asp | GTC | - | - | No |
| 1104887..1104959 | Gln | CTG | - | - | No |
| 1432100..1432172 | Gln | TTG | - | - | No |
| 1178938..1179030 | Glu | CTC | - | - | No |
| 1376626..1376715 | Glu | TTC | - | - | No |
| 1247049..1247124 | Gly | CCC | - | - | No |
| 1431771..1431846 | Gly | GCC | - | - | No |
| 1479146..1479221 | Gly | TCC | - | - | No |
| 1638129..1638202 | His | GTG | - | - | No |
| 996201..996286 | Ile | GAT | 996239 | 996250 | Yes |
| 1057784..1057882 | Leu | CAA | 1057843 | 1057829 | Yes |
| 1186783..1186867 | Leu | CAG | - | - | - |
| 111325..111409 | Leu | GAG | - | - | No |
| 1188894..1188993 | Leu | TAA | 1188953 | 1188939 | Yes |
| 1162261..1162345 | Leu | TAG | - | - | No |
| 1678998..1679093 | Lys | CTT | 1679036 | 1679057 | Yes |
| 1697966..1698062 | Lys | TTT | 1698004 | 1698026 | Yes |
| 1365026..1365117 | Met | CAT | 1365065 | 1365081 | Yes |
| 1632832..1632934 | Met | CAT | 1632871 | 1632895 | Yes |
| 1185332..1185405 | Met | CAT | - | - | No |
| 1230694..1230767 | Phe | GAA | - | - | No |
| 1486173..1486248 | Pro | CGG | - | - | No |
| 1011465..1011561 | Pro | GGG | 1011505 | 1011525 | Yes |
| 1760144..1760219 | Pro | TGG | - | - | No |
| 1184871..1184978 | Ser | CGA | 1184908 | 1184931 | Yes |
| 1759153..1759237 | Ser | GCT | - | - | No |
| 1631494..1631577 | Ser | GGA | - | - | No |
| 1476812..1476896 | Ser | TGA | - | - | No |
| 1610286..1610373 | Thr | CGT | 1610325 | 1610337 | Yes |
| 2207684..2207758 | Thr | GGT | - | - | No |
| 1105124..1105213 | Thr | TGT | 1105163 | 1105177 | Yes |
| 1736117..1736255 | Trp | CCA | 1736155 | 1736219 | Yes |
| 1183357..1183445 | Tyr | GTA | 1183397 | 1183409 | Yes |
| 1698229..1698303 | Val | CAC | - | - | No |
| 1589773..1589847 | Val | GAC | - | - | No |
| 1179203..1179277 | Val | TAC | - | - | No |

Table S8. tRNA genes of *S. islandicus* LAL14/1

| **Strain** | **Atypical coding genes %**  **by genome** | **Fraction of atypical coding genes in large variable region** | **% of atypical genes situated in large variable region** |
| --- | --- | --- | --- |
| LAL14/1 | 21,4% | 49,0% | 55,2% |
| REY15A | 19,9% | 46,4% | 55,7% |
| HVE10/4 | 18,9% | 50,9% | 63,2% |
| LD85 | 22,6% | 47,4% | 48,4% |
| YG5714 | 22,3% | 51,1% | 51,1% |
| YN1551 | 21,5% | 50,7% | 62,5% |
| M1425 | 12,4% | 35,7% | 59,9% |
| LS215 | 17,0% | 42,3% | 59,9% |
| M1627 | 14,4% | 40,4% | 68,8% |
| M164 | 16,0% | 40,9% | 61,2% |

**Table S9. CAG frequency and distribution in 10 *S. islandicus* genomes**

| **Replication origin** | **OriC-1** | **OriC-2** | **OriC-3** |
| --- | --- | --- | --- |
| **Gene linked** | ***cdc6-1*** | ***cdc6-3*** | ***whiP*** |
| strains |  |  |  |
| *S. solfataricus* P2 | 220kb | 2Mb | 740kb |
| *S. solfataricus* 98/2 | 1,15Mb | 800pb | 1,65Mb |
| *S. tokodaii* | 322kb | 2,16Mb | 1,25Mb |
| *S. acidocaldarius* | 578kb | 100pb | 1,19Mb |
| *S. islandicus* LAL 14/1 | 1,59Mb | 800pb | 1,15Mb |
| *S. islandicus* HVE 10/4 | 1,75Mb | 800pb | 1,30Mb |
| *S. islandicus* REY15A | 1,6Mb | 800pb | 1,16Mb |
| *S. islandicus* M16.4 | 1,73Mb | 800pb | 1,26Mb |
| *S. islandicus* M16.27 | 1,83Mb | 800pb | 1,36Mb |
| *S. islandicus* M14.25 | 1,75Mb | 800pb | 1,280Mb |
| *S. islandicus* YG57.14 | 1,76Mb | 800pb | 1,25Mb |
| *S. islandicus* YN15.51 | 863kb | 800pb | 1,31Mb |
| *S. islandicus* LD8.5 | 1,82Mb | 800pb | 1,29Mb |
| *S. islandicus* LS2.15 | 1,81Mb | 800pb | 1,34Mb |

Table S10. Positions of the three origins of replication *oriC* in different *Sulfolobus* genomes.

| ***S. islandicus***  **strains** | **Number of**  ***vapBC* loci** | **Number of**  ***HEPN-NT** loci** |
| --- | --- | --- |
| LAL 14/1 | 15 | 5 |
| HVE 10/4 | 18 | 5 |
| REY 15A | 17 | 6 |
| LD8.5 | 21 | 6 (3) |
| LS2.15 | 24 | 6 (1) |
| M14.25 | 21 | 6 (1) |
| M16.4 | 21 | 6 (1) |
| M16.27 | 21 | 6 (1) |
| YG57.14 | 20 | 6 |
| YN15.51 | 19 | 6 (1) |

**Table S11. *vapBC* and *HEPN-NT* loci in *S. islandicus.* The digits in brackets indicate the number of loci carrying at least one disrupted ORF; these copies of HEPN-HT are considered to be non-functional**

|  |  | REY 15A | LS85 | M1425 | LS215 | YG5714 | YN1551 | M1627 | M164 | HVE10/4 | LAL14/1 | Genomic region |
| --- | --- | --- | --- | --- | --- | --- | --- | --- | --- | --- | --- | --- |
| Family I | HEPN | SiRe_0947 | LD85_1191 | M1425_1074 | LS215_1171 | YG5714_1064 | YN1551_1798 | M1627_1138 | M164_1063 | SiH_1034 | SiL_0948 | Conserved region |
|  | NT | SiRe_0948 | LD85_1190 | M1425_1073 | LS215_1170 | YG5714_1063 | YN1551_1799 | M1627_1137 | M164_1062 | SiH_1035 | SiL_0947 |  |
| Family II | HEPN | SiRe_0938 | LD85_1187 | M1425_1070 | LS215_1167 | YG5714_1060 | YN1551_1802 | M1627_1134 | M164_1059 | SiH_1027 | SiL_0944 |  |
|  | NT | SiRe_0939 | LD85_1188 | M1425_1071 | LS215_1168 | YG5714_1061 | YN1551_1801 | M1627_1135 | M164_1060 | SiH_1028 | SiL_0945 |  |
|  | HEPN | SiRe_0917 |  |  |  | YG5714_2031 | YN1551_0221 |  |  |  |  |  |
|  | NT | SiRe_0918 |  |  |  | YG5714_2030 | YN1551_0222 |  |  |  |  |  |
|  | HEPN | SiRe_0458 | LD85_0337 | M1425_0330 | LS215_0355 | YG5714_0333 | YN1551_2725 | M1627_0330 | M164_0352 | SiH_0331 | SiL_0488 | Variable region |
|  | NT | SiRe_0459 | LD85_0336 | M1425_0329 | LS215_0354 | YG5714_0332 | YN1551_2526 | M1627_0329 | M164_0351 | SiH_0330 | SiL_0487 |  |
|  | HEPN | SiRe_0924 | LD85_0452* | M1425_0753* | LS215_0865 | YG5714_0920 | YN1551_1959 | M1627_0758* | M164_0795* | SiH_1009 | SiL_1923 |  |
|  | NT | SiRe_0923 | LD85_0451 | M1425_0752 | LS215_0864 | YG5714_0919 | YN1551_1960 | M1627_0757 | M164_0794 | SiH_1008 | SiL_1922 |  |
|  | HEPN | SiRe_0902 | LD85_1061* | M1425_0454 | LS215_0536 | YG5714_1044 | YN1551_1820 | M1627_1098 | M164_1041 | SiH_0561 | SiL_0783 |  |
|  | NT | SiRe_0903 | LD85_1060 | M1425_0455 | LS215_0535 | YG5714_1043 | YN1551_1819 | M1627_1099 | M164_1040 | SiH_0562 | SiL_0782 |  |
|  | HEPN | SiRe_0731 | LD85_0839* | M1425_0456 | LS215_0829* | YG5714_0965 | YN1551_0892* | M1627_0468 | M164_0490 |  |  |  |
|  | NT | SiRe_0730 | LD85_0838 | M1425_0458 | LS215_0828 | YG5714_0966 | YN1551_0893 | M1627_0469 | M164_0491 |  |  |  |
|  | HEPN |  | LD85_0841 |  |  |  | YN1551_2062 | M1627_0851 |  |  |  |  |
|  | NT |  | LD85_0842 |  |  |  | YN1551_2063 | M1627_0852 |  |  |  |  |

**Table S12. Diversity of the HEPN-NT gene pairs in 10 *S. islandicus* genomes**

* truncated version of corresponding genes

| ***Sulfolobus* species** | ***ups* operons** | | | | |
| --- | --- | --- | --- | --- | --- |
| **SSP2** | SSO0117 | SSO0118 | SSO0119 | SSO0120 | SSO0121 |
| **SS98/2** | Ssol98_02032 | Ssol98_02037 | Ssol98_02042 | Ssol_1095 | Ssol98_02052 |
| **LAL 14.1** | SiL_1862 | SiL_1861 | SiL_1860 | SiL_1859 | SiL_1858 |
| **M.14.25** | M1425_2008 | M1425_2007 | M1425_2006 | M1425_2005 | M1425_2004 |
| **M.16.4** | M164_2015 | M164_2014 | M164_2013 | M164_2012 | M164_2011 |
| **M.16.27** | M1627_2086 | M1627_2085 | M1627_2084 | M1627_2083 | M1627_2082 |
| **HVE 10/4** | SiH_1954 | SiH_1953 | SiH_1952 | SiH_1951 | SiH_1950 |
| **L.S.2.15** | LS215_2173 | LS215_2172 | LS215_2171 | LS215_2170 | LS215_2169 |
| **L.D.8.5** | LD85_2273 | LD85_2272 | LD85_2271 | LD85_2270 | LD85_2269 |
| **REY15A** | SiRe_1882 | SiRe_1881 | SiRe_1880 | SiRe_1879 | SiRe_1878 |
| **Y.G.57.14** | YG5714_2134 | YG5714_2133 | YG5714_2132 | YG5714_2131 | YG5714_2130 |
| **Y.N.15.51** | YN1551_0785 | YN1551_0786 | YN1551_0787 | YN1551_0788 | YN1551_0789 |
| ***S.tokodaii*** | ST1400 | ST1399 | ST1398 | ST1397 | ST1396 |
| ***S.acidocaldarius*** | not annotated | Saci_1496 | Saci_1495 | Saci_1494 | Saci_1493 |
| ***M.sedulla*** | Msed_2107 | Msed_1193 | Msed_2105 | Msed_2104 | Msed_2103 |

**Table S13. Positions the operons *ups* coding for the pili of type IV in *Sulfolobales***

|  |  | ***S. islandicus* strains** | | | | | |
| --- | --- | --- | --- | --- | --- | --- | --- |
|  |  | **HVE 10/4*** | | **REY 15A*** | | **LAL 14/1** | |
| Family | Element | Intact orf | Total | Intact orf | Total | Intact orf | Total (variable) |
| *IS*1 | ISC796 | 1 | 4 | 0 | 5 | 1 (0) | 9 (2) |
| *IS*5 | ISC1058 | 0 | 1 | 0 | 2 | 0 | 3 (0) |
| *IS*5 | ISC1234 | 5 | 7 | 6 | 8 | 0 | 0 |
| *IS*6 | ISSis1 | 1 | 2 | 1 | 2 | 0 | 0 |
| *IS*6 | ISSto2 | 4 | 5 | 2 | 4 | 0 | 2 (1) |
| *IS*110 | ISC1190 | 0 | 1 | 1 | 3 | 0 | 3 (0) |
| *IS*110 | ISC1229 | 9 | 10 | 2 | 4 | 1 (0) | 2 (1) |
| *IS*110 | ISSto4 | 0 | 0 | 0 | 0 | 1 (1) | 1 (1) |
| *IS*200/605 | ISC1733 | 2 | 2 | 8 | 8 | 4 (2) | 4 (2) |
| *IS*256 | ISC1332 | 1 | 1 | 1 | 1 | 0 | 1 (1) |
| *IS*605 | OrfB | 17 | 18 | 18 | 19 | 10 | 12 (10) |
| *IS*607 | ISC1921 | 0 | 0 | 1 | 1 | 0 | 0 |
| *IS*630 | ISC1048 | 0 | 12 | 0 | 10 | 0 | 8 (4) |
| *IS*630 | ISC1078 | 0 | 1 | 0 | 1 | 0 | 0 |
| *IS*630 | ISC1395 | 0 | 0 | 1 | 2 | 0 | 2 (2) |
| *IS*H3 | ISC1200 | 3 | 7 | 11 | 22 | 0 | 3 (2) |
| *IS*CNY | ISC1205 | 2 | 4 | 0 | 3 | 0 | 3 (3) |
| ISL3 | ISC1043 | 0 | 1 | 0 | 1 | 0 | 0 |
| **Total** |  | **45** | **76** | **52** | **96** | **7** | **53** |

**Table S14. Comparison of the *IS* content of the genomes of three *S. islandicus* strains isolated from Iceland.**

* data from Guo et al*., J Bact* 2011;193(7):1672-1680.

|  | *HVE 10/4 | *REY15A | LAL 14/1 | LD8.5 | LS2.15 | M14.25 | M16.27 | M16.4 | YG57.14 | YN15.51 |
| --- | --- | --- | --- | --- | --- | --- | --- | --- | --- | --- |
| SM3A | 2(1) | 2(1) | 11(7) | 3(2) | 3(2) | 3(2) | 4(4) | 4(3) | 3(2) | 2(1) |
| SMN1 | 9(8) | 7(7) | 9(9) | 0 | 0 | 2(2) | 1 | 5(4) | 0 | 0 |

**Table S15. MITEs distribution in *S. islandicus*.** In brackets the number of MITEs situated in the variable region. * - as published by Guo et al*., J Bact* 2011;193(7):1672-1680.

| **Spacers** | **Putative targets** | **Targeted ORF** | **Length of spacer *vs*. target** | **Number of mismatches** |
| --- | --- | --- | --- | --- |
| **1_11** | **SIRV1 VIII** | **ORF98** | **45/45** | **0** |
| 1_36 | SIRV2 | ORF119c | 39/39 | 2 |
|  | SIRV1 XX | ORF119 | 39/39 | 3 |
|  | SIRV1 VIII | ORF117 | 39/39 | 3 |
| 1_66 | SIRV1 XX | ORF112 | 41/41 | 4 |
|  | SIRV1 VIII | ORF112 | 41/41 | 4 |
| 1_77 | SSV2 | ORF72 | 45/45 | 1 |
| 1_83 | SIRV2 | ORF436 | 38/44 | 3 |
|  | SIRV1 XX | ORF440 | 38/44 | 4 |
|  | SIRV1 VIII | ORF440 | 38/44 | 4 |
| 1_85 | STIV2 | STIV2_B265 | 37/40 | 3 |
| 1_86 | SSV7 | SSV7_D336 | 40/41 | 2 |
|  | SSV2 | ORF328 | 40/41 | 2 |
| 2_3 | SIRV2 | - | 41/46 | 5 |
| 2_6 | pING1 | ORF1042 | 40/42 | 2 |
|  | pSOG2 | ORF1094 | 42/42 | 3 |
| 2_13 | STIV2 | STIV2_F98 | 39/39 | 1 |
| 2_38 | SSV2 | ORF328 | 40/40 | 1 |
| 2_40 | pXZ1 | ORF82 | 38/40 | 2 |
| 2_58 | SIRV1 | ORF1070 | 40/40 | 1 |
|  | SIRV1 XX | ORF1070 | 40/40 | 5 |
|  | SIRV2 | ORF1070 | 40/40 | 5 |
| 2_64 | STIV2 | STIV2_C141  STIV2_E65 | 34/39 | 1 |
| 2_67 | pLD8501 | LD85_3160 | 41/42 | 2 |
|  | L.D.8.5 | LD85_1584 | 41/42 | 2 |
|  | pAH1 | ORF78 | 41/42 | 2 |
|  | M.16.27 | M1627_1565 | 42/42 | 3 |
|  | 98/2 | Ssol_1586 | 42/42 | 4 |
|  | P2 | - | 42/42 | 4 |
| 2_83 | M.14.25 | M1425_1876 | 41/41 | 4 |
|  | M.16.4 | M164_1349 | 41/41 | 5 |
|  | Y.N.15.51 | YN1551_0201 | 41/41 | 5 |
|  | Y.G.57.14 | YG5714_2694 | 41/41 | 5 |
| 2_88 | SIRV1 XX | - | 35/39 | 1 |
|  | SIRV1 | - | 35/39 | 1 |
| 2_92 | pARN4 | - | 40/42 | 3 |
| 2_93 | **pARN3** | **ORF609** | **41/41** | **0** |
| 2_98 | SIRV1 XX | ORF1070 | 41/41 | 1 |
|  | SIRV2 | ORF1070 | 41/41 | 2 |
| 2_112 | SIRV1 VIII | ORF356 | 41/42 | 2 |
|  | SIRV1 XX | ORF356 | 41/42 | 2 |
|  | SIRV2 | ORF356 | 41/42 | 3 |
| 2_113 | M.16.45 | CRISPR region | 40/41 | 2 |
|  | M .16.43 | CRISPR region | 40/41 | 2 |
| 3_8 | SIRV1 XX | ORF335 | 34/34 | 2 |
|  | SIRV1 VIII | ORF335 | 34/34 | 2 |
| 4_1 | SIRV1 XX | ORF105 | 33/33 | 1 |
|  | SIRV1 | ORF90 | 33/33 | 1 |
| 4_5 | SIRV1 XX | ORF74 | 36/36 | 2 |
|  | SIRV VIII | ORF84a | 36/36 | 2 |
|  | LS2.15 | CRISPR | 34/36 | 2 |
| 4_6 | SIRV2 | ORF309/158a | 36/36 | 2 |
|  | SIRV1 XX | ORF417/158b | 36/36 | 3 |
|  | SIRV VIII | ORF417/158b | 36/36 | 3 |
| 4_17 | SIRV1 XX | ORF356 | 36/36 | 1 |
|  | SIRV1 VIII | ORF356 | 36/36 | 1 |
|  | SIRV2 | ORF356 | 35/36 | 2 |
| 5_1 | **SIRV1 XX** | **ORF440** | **37/37** | **0** |
|  | **SIRV1 VIII** | **ORF440** | **37/37** | **0** |
|  | SIRV-2 | ORF436 | 37/37 | 3 |
| 5_2 | SIRV2 | ORF56b/119c | 38/38 | 2 |
| 5_3 | SIRV2 | ORF156 | 32/35 | 1 |
|  | SIRV1 XX | ORF156 | 33/35 | 2 |
| 5_6 | SIRV2 | ORF436 | 36/39 | 1 |
|  | SIRV1 XX | ORF440 | 35/39 | 1 |
|  | SIRV1 VIII | ORF440 | 35/39 | 1 |
| 5_7 | SIRV1 XX | ORF105 | 33/33 | 1 |
|  | SIRV1 VIII | ORF105 | 33/33 | 1 |
|  | SIRV2 | ORF102 | 33/33 | 2 |

**Table S16. Spacers/targets correspondence for the CRISPRs of *S. islandicus* LAL14/1.** For the spacers shorter than targets only the spacers corresponding to 85% or more of the putative target length retained. Spacers with 100% of correspondence to the targets are in bold.

**
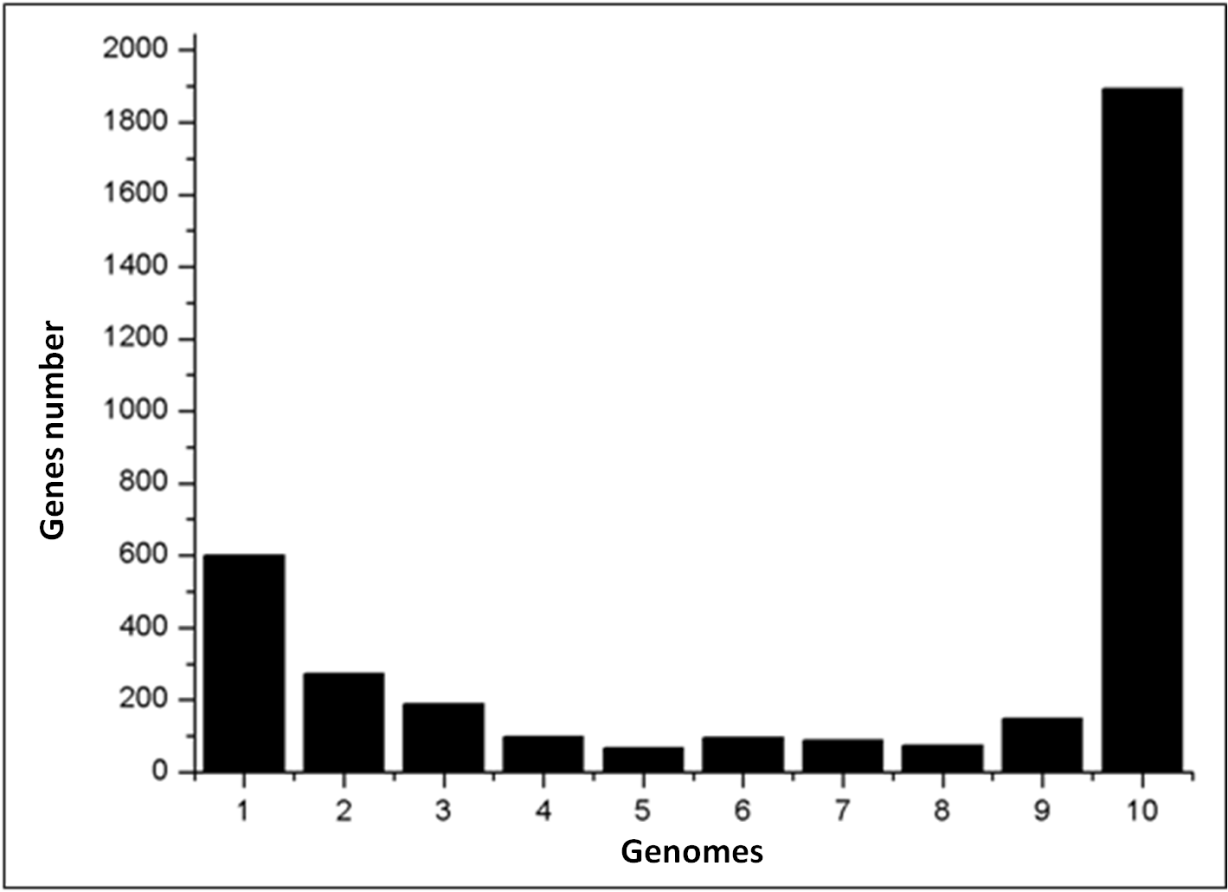
**

**Figure S1. *S. islandicus* pan genome analysis. The histogram indicates the number of genes shared by indicated number of *S. islandicus* genomes. Plot 1 – unique genes present in only one from 10 genomes (*singletons*); plot 10 – number of ubiquitous genes shared by all *S. islandicus* (core genome).**

**Figure S2. Singleton distribution in ten *S. islandicus* strains.**

**
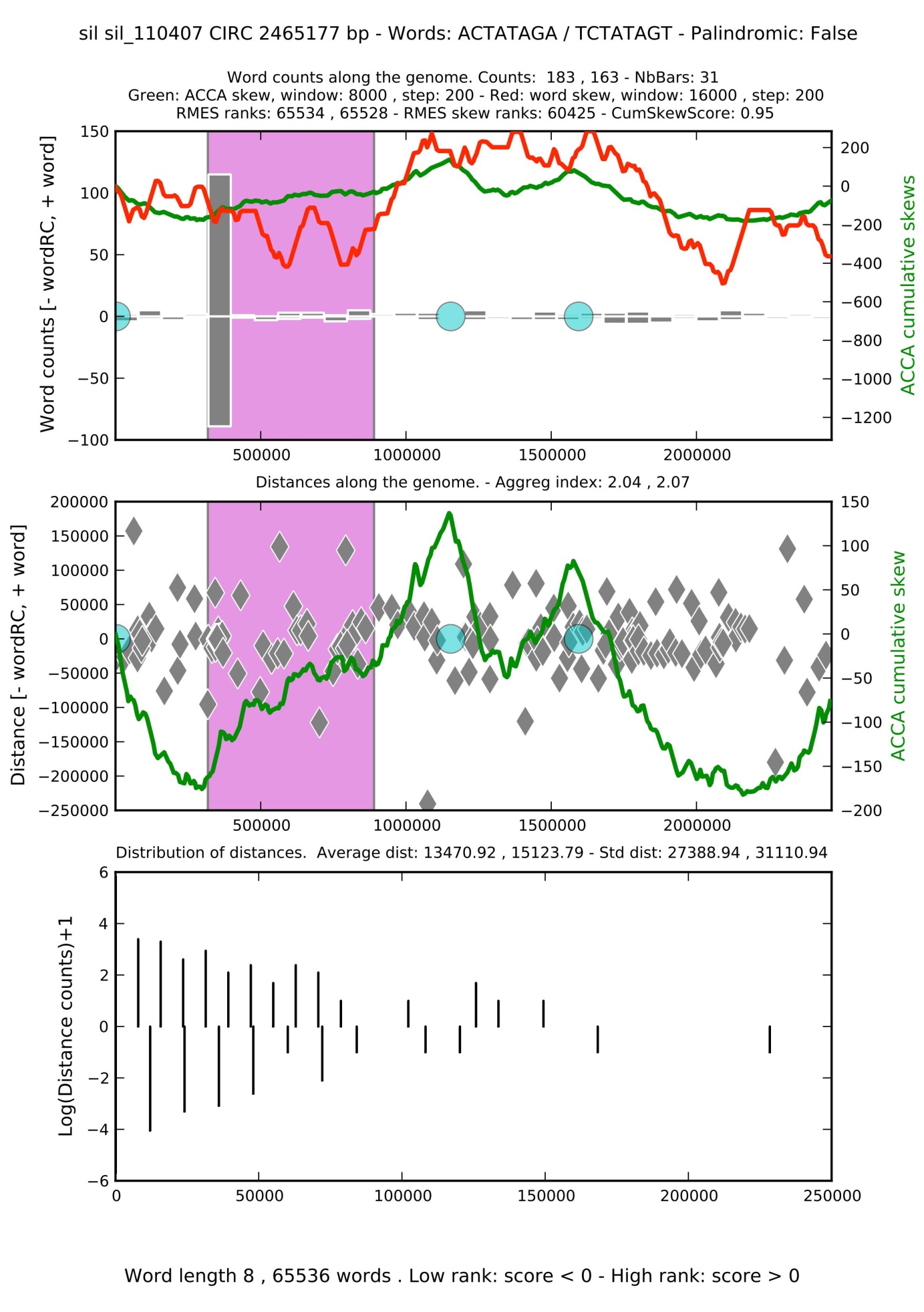
**

**Figure S3. Representation of the word ACTATAGA in the LAL14/1 genome.** The word counts are indicating along the genome sequence by gray bars. Red curve represents the skew of the word ACTATAGA; green curve shows the skew of ACCA indicating the positions of the replication origins, also marked as circles. The hyper-variable region is represented by a pink rectangle.

**
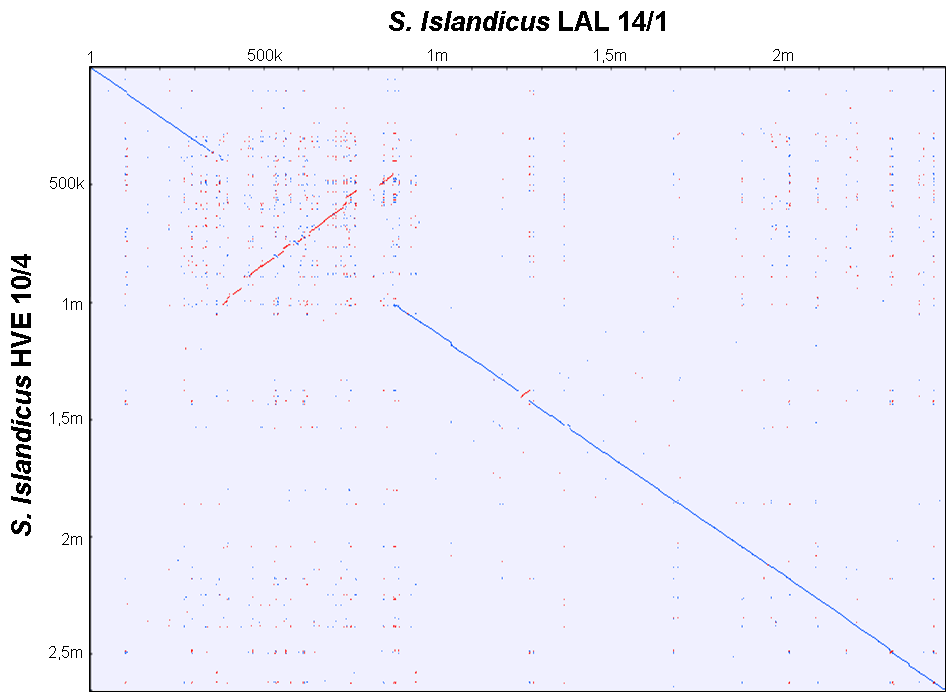
**

**
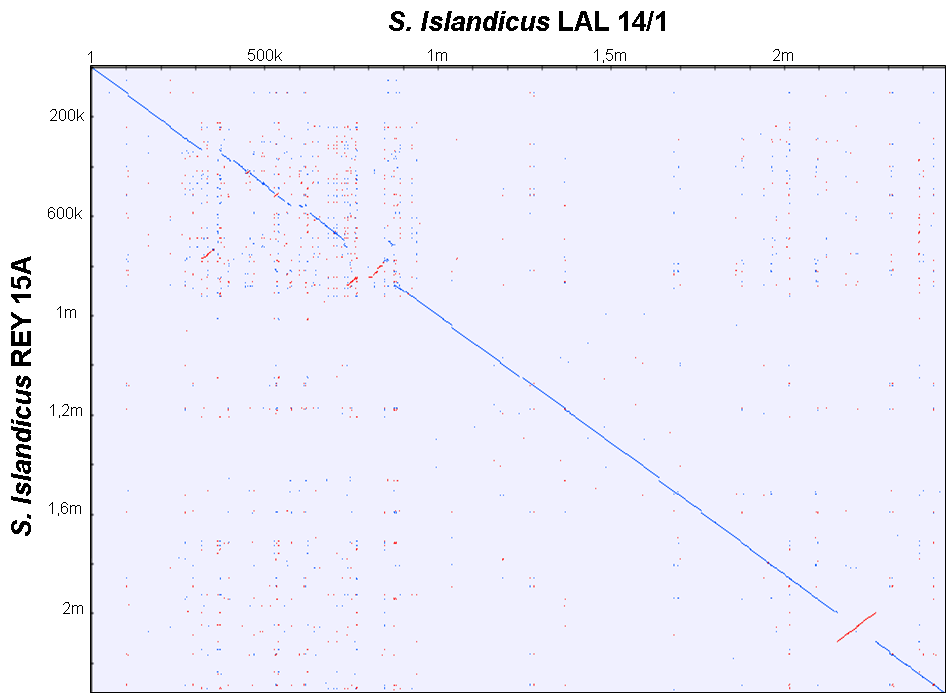
**

Figure S4. Dot-plot comparison of (A) *S. islandicus* LAL14/1 vs HVE10/4 and (B) LAL14/1 vs REY15A. Commented in Table S4.

**Figure S5.** **Remnants of fuselloviral genomes in different *S. islandicus* strains.** Equivalent genomic loci of selected *S. islandicus* strains encompassing remnants of SSV-like proviruses are aligned; linearized genome map of fusellovirus SSV2 is also included in the alignment. Pairwise tblastx hits between the corresponding loci are illustrated by different shades of grey (the identity scale is included in the figure). Genome accession numbers of the depicted *S. islandicus* strains are provided below the corresponding strain names. Homologues of fuselloviral genes, blue arrows; non-viral genes, red arrows; tRNA^Thr^[GGT] genes, green rectangles.

**
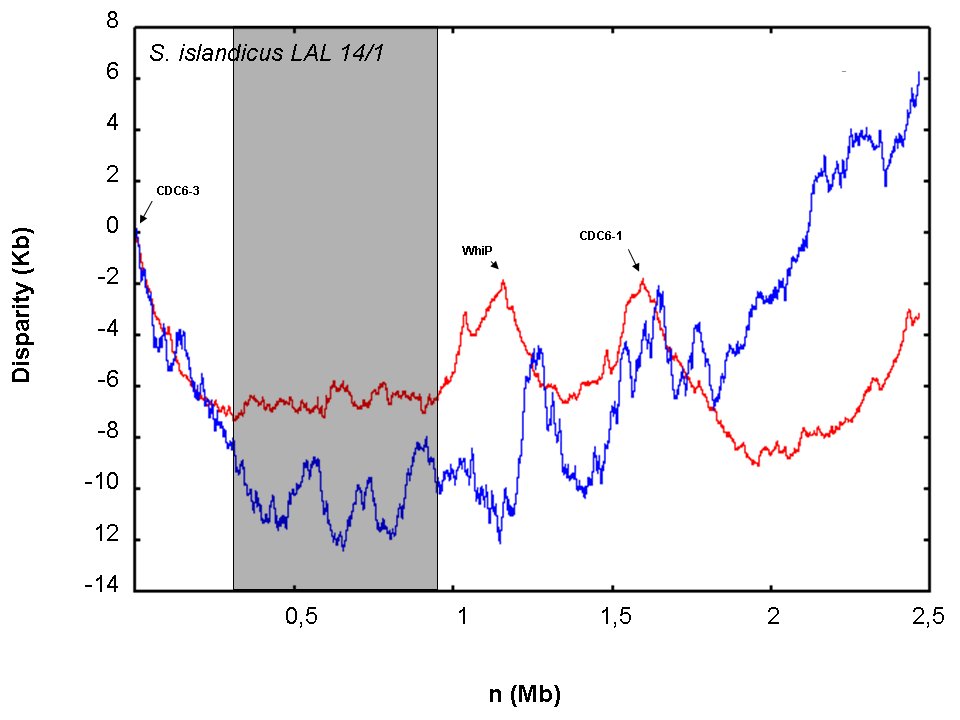
**

**A.**

**
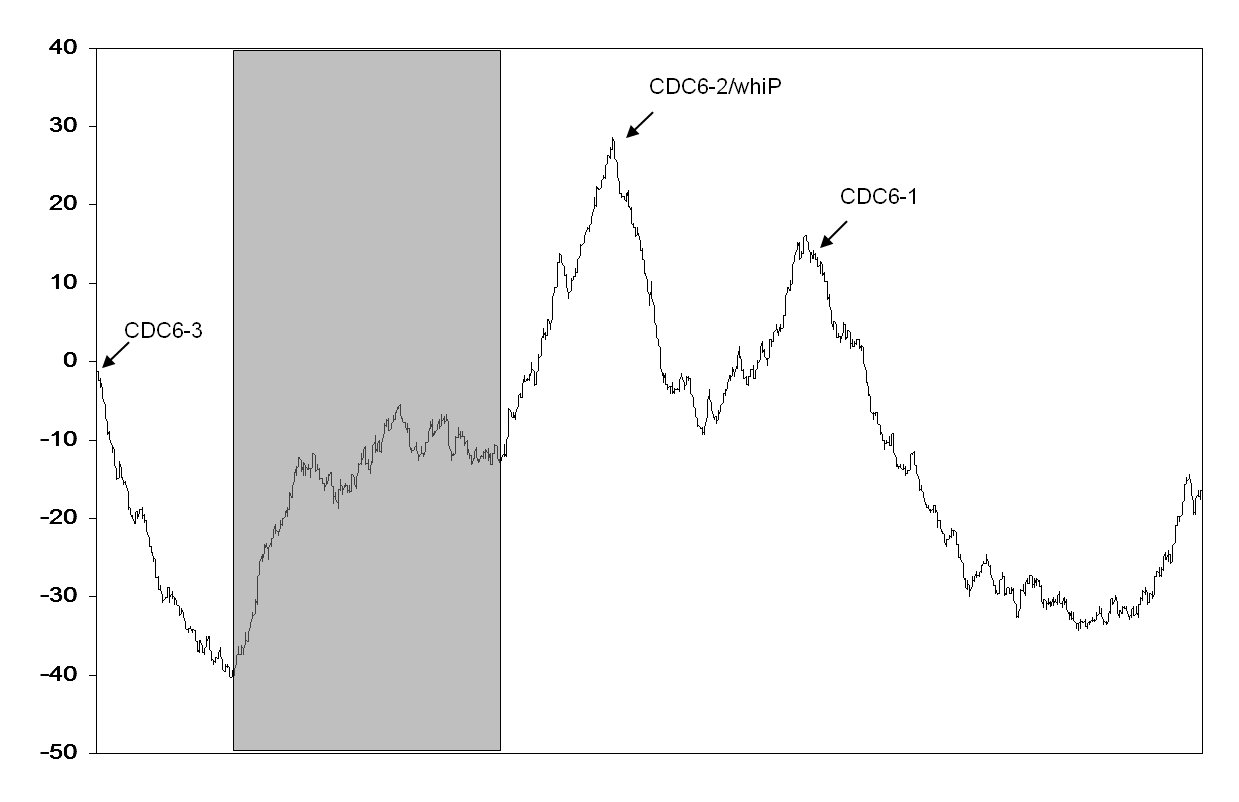
**

**B.**

**Figure S6. Identification of origins of replication in *S. islandicus* LAL14/1 genome by (A) approach Z-curve; (B) ACCA plot.** Arrows indicate the positions of some conserved genes linked to three *oriC*. The position of long variable region is indicated by shadow rectangle**.**

**
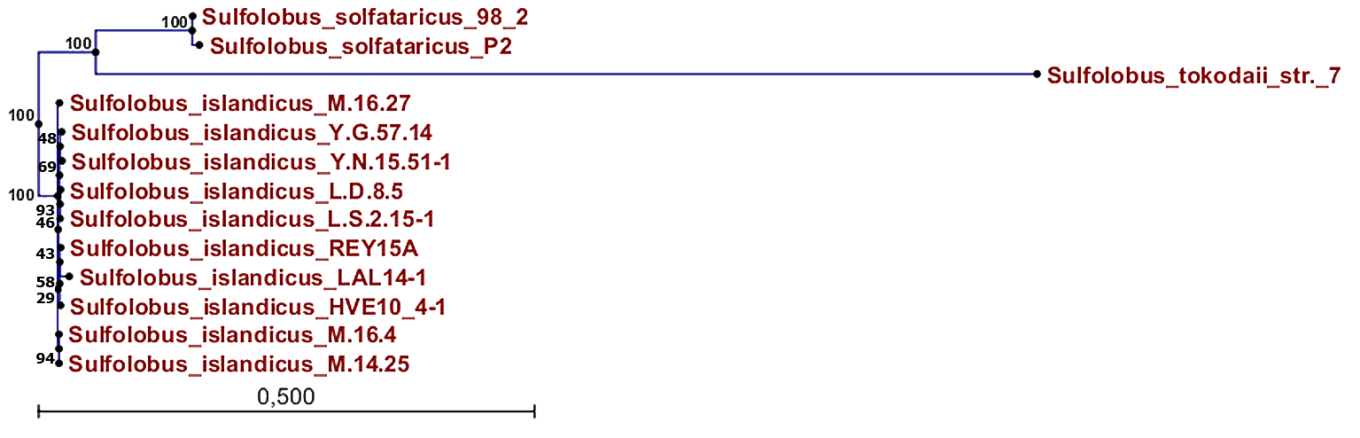
**

**Figure S7. Phylogenetic tree of the proteins of Ups family in *Sulfolobales***
